# Ligand-based targeting of c-kit using engineered γδ T cells as a strategy for treating acute myeloid leukemia

**DOI:** 10.3389/fimmu.2023.1294555

**Published:** 2023-11-13

**Authors:** Gianna M. Branella, Jasmine Y. Lee, Jennifer Okalova, Kiran K. Parwani, Jordan S. Alexander, Raquel F. Arthuzo, Andrew Fedanov, Bing Yu, David McCarty, Harrison C. Brown, Shanmuganathan Chandrakasan, Brian G. Petrich, Christopher B. Doering, H. Trent Spencer

**Affiliations:** ^1^ Department of Pediatrics, Emory University School of Medicine, Atlanta, GA, United States; ^2^ Graduate Division of Biological and Biomedical Sciences, Laney Graduate School, Emory University, Atlanta, GA, United States; ^3^ Aflac Cancer and Blood Disorders Center, Children’s Healthcare of Atlanta, Atlanta, GA, United States; ^4^ Molecular Systems Pharmacology Program, Graduate Division of Biological and Biomedical Sciences, Laney Graduate School, Emory University, Atlanta, GA, United States; ^5^ Expression Therapeutics, Inc., Tucker, GA, United States

**Keywords:** acute myeloid leukemia (AML), gamma delta (γδ) T cells, ligand-based therapeutics, chimeric antigen receptor (CAR), secreted bispecific T cell engager, stem cell factor (SCF), c-kit (CD117)

## Abstract

The application of immunotherapies such as chimeric antigen receptor (CAR) T therapy or bi-specific T cell engager (BiTE) therapy to manage myeloid malignancies has proven more challenging than for B-cell malignancies. This is attributed to a shortage of leukemia-specific cell-surface antigens that distinguish healthy from malignant myeloid populations, and the inability to manage myeloid depletion unlike B-cell aplasia. Therefore, the development of targeted therapeutics for myeloid malignancies, such as acute myeloid leukemia (AML), requires new approaches. Herein, we developed a ligand-based CAR and secreted bi-specific T cell engager (sBite) to target c-kit using its cognate ligand, stem cell factor (SCF). c-kit is highly expressed on AML blasts and correlates with resistance to chemotherapy and poor prognosis, making it an ideal candidate for which to develop targeted therapeutics. We utilize γδ T cells as a cytotoxic alternative to αβ T cells and a transient transfection system as both a safety precaution and switch to remove alloreactive modified cells that may hinder successful transplant. Additionally, the use of γδ T cells permits its use as an allogeneic, off-the-shelf therapeutic. To this end, we show mSCF CAR- and hSCF sBite-modified γδ T cells are proficient in killing c-kit^+^ AML cell lines and sca-1^+^ murine bone marrow cells *in vitro*. *In vivo*, hSCF sBite-modified γδ T cells moderately extend survival of NSG mice engrafted with disseminated AML, but therapeutic efficacy is limited by lack of γδ T-cell homing to murine bone marrow. Together, these data demonstrate preclinical efficacy and support further investigation of SCF-based γδ T-cell therapeutics for the treatment of myeloid malignancies.

## 1 Introduction

Adoptive cell therapy (ACT), such as chimeric antigen receptor (CAR) T therapy, has significantly advanced the treatment of B cell malignancies, leading to the FDA approval of multiple cellular products (1–6). However, ACT for other leukemias, like acute myeloid leukemia (AML), do not share the same success. Current treatment regimens for AML consist of genotoxic chemotherapeutics (7) that have severe toxicities in the bone marrow (8), with approximately 30% of children relapsing (9–12). This leads to a drop in survival from 70% to a mere 20% (9, 13), highlighting the need for new treatment options.

Unfortunately, there are a lack of known leukemia-specific, cell-surface antigens that distinguish healthy from malignant myeloid cells (14). This makes the translation of traditional cell-based immunotherapies with long-term persistence challenging in this setting, as myeloid depletion cannot be managed like B-cell aplasia. However, as hematopoietic stem cell transplantation (HSCT) is considered curative for AML, it may be important to take advantage of novel therapeutics that display off-tumor toxicities in the hematopoietic compartment that can be corrected with HSCT. In fact, toxicity within the bone marrow may prove advantageous to dually prepare the patient for transplant and target residual disease congruently.

We have developed receptor-directed, ligand-based ACT to target malignant myeloid tissue through interaction with c-kit using its cognate ligand, stem cell factor (SCF). Up to 90% of AML patients have c-kit expression correlating with poor prognosis and resistance to chemotherapy (15, 16), making this receptor an attractive target for malignant myeloid cell and leukemic stem cell (LSC) ablation. In fact, we (17) and others (18–22) have shown the continued use of c-kit as a target for non-genotoxic conditioning due to its expression on hematopoietic stem cells (HSCs). These therapies have proven safe for use in preclinical murine models with toxicities only observed within the hematopoietic compartment (17) despite limited c-kit expression on some organs outside of the hematopoietic compartment, like the cerebellum, female reproductive organs, and lungs. Clinically, c-kit is the target of monoclonal antibody briquilimab, which has shown promise in treating patients with AML and myelodysplastic syndromes (MDS) in a Phase 1 clinical trial (NCT04429191), Fanconi anemia in a Phase 1/2 clinical trial (NCT04784052), severe combined immunodeficiency (SCID) in a Phase 1/2 clinical trial (NCT02963064), and sickle cell disease in a Phase 1/2 clinical trial (NCT05357482) with no treatment-related adverse events in 130 dosed subjects as of late September 2023 (23, 24).

Herein, we utilize ligand-based, c-kit-directed, CAR- and secreted bispecific T-cell engager (sBite)-modified T cells as a therapeutic for the treatment of AML. Traditionally, CARs and bispecific antibodies are designed with single chain variable fragments (scFvs) as their antigen-binding domains that specifically redirect T cells to kill cancer cells without the need for antigen processing and presentation by the human leukocyte antigen (HLA) (25). However, these designs are not without flaws. Primarily, scFv molecules are prone to aggregation and produce higher order complexes through intermolecular variable heavy (V_H_) and variable light (V_L_) association, resulting in tonic signaling and thereby lower therapeutic efficacy (26–28). Our proposed ligand-based design circumvents these issues.

Furthermore, we employ CAR- and sBite-modified γδ T cells as a cytotoxic alternative to αβ T cells. γδ T cells contribute to graft-versus-leukemia (GvL) reactions to enhance cancer eradication through the recognition of stress antigens expressed by leukemia cells (29–31). Additionally, as γδ T cells recognize antigen independent of HLA, they can be transplanted across HLA barriers in the presence of immunosuppression, thus creating opportunity for an off-the-shelf therapeutic. Importantly, we (31–36) and others (37) have optimized the use of γδ T cells and can expand these cells *ex vivo* using a novel serum-free protocol resulting in a cellular product of highly purified Vγ9Vδ2 T cells with low percentages of NK cells and negligible αβ T cell contamination. The chemokine receptor expression and exhaustion profiles of these cells have been reported (35, 38)., in addition to the phenotype of the contaminating NK cells within our cellular product (38).

These studies establish a ligand-based cell therapy strategy using engineered γδ T cells as a cytotoxic alternative to traditional ACT for the treatment of AML. Non-modified allogeneic γδ T cells expanded under our Good Manufacturing Practice (GMP)-compliant serum-free protocol are currently under clinical investigation in a Phase 1 trial for the treatment of relapsed/refractory neuroblastoma (NCT05400603). In the context of myeloid malignancies, we have previously demonstrated non-modified γδ T cells have enhanced cytotoxicity against AML cell lines in combination with chemotherapeutic agents that upregulate stress antigens expressed by AML cells (31). More recently, we showed our efforts in optimizing and utilizing expanded γδ T cells transiently transfected with mRNA encoding a CD19 CAR for the treatment of B-cell malignancies (39). Here, we expand our repertoire by investigating the transient modification of γδ T cells using a ligand-based CAR and sBite. These studies provide the basis for further investigation into the use of ligand-based γδ T cell therapies.

## 2 Materials and methods

### 2.1 c-kit and NKG2D ligand expression on healthy donor and patient samples

The St. Jude Cloud (pecan.stjude.cloud) was queried for c-kit expression using transcriptomic data from 2,480 pediatric patients (40, 41). The Human Protein Atlas (v22.proteinatlas.org) was queried for c-kit expression in healthy tissues and cell lines by RNA sequencing (42). The R2 Genomics Analysis and Visualization Platform (https://r2.amc.nl) was used to query multiple datasets using the Megasampler R2 module for expression of c-kit, ULBP1, ULBP2, and MICA/MICB by RNA sequencing. The u133a chip with MAS5.0 normalization was used exclusively. Datasets utilized in this study were as follows: PBMC (Novershtern, 211 samples), HSC (OGIC, 21 samples), and AML (Bohlander, 422 samples and Delwel, 293 samples).

### 2.2 Cell lines

The NOMO-1, Kasumi-1, KG-1, and MOLM-13 cell lines were kindly gifted by the laboratory of Dr. Douglas Graham (Emory University, Atlanta, GA, USA). The CMK cell line was kindly provided by the laboratory of Dr. Brian Petrich (Emory University, Atlanta, GA, USA). The Jurkat, K562, and 697 cell lines were obtained from the American Type Culture Collection (Manassas, VA, USA). NOMO-1, K562, 697, KG-1, and Jurkat cells were cultured in RPMI-1640 with L-glutamine (Corning CellGro, Manassas, VA, USA) supplemented with 10% fetal bovine serum (FBS; R&D Systems, Minneapolis, MN, USA) and 1% Penicillin-Streptomycin (Cytiva, Marlborough, MA, USA). The CMK, Kasumi-1, and MOLM-13 cell lines were cultured under the same conditions using 20% FBS.

### 2.3 Design and cloning of mSCF CAR and hSCF sBite

mSCF CAR and hSCF sBite DNA sequences were cloned into vectors containing the necessary components for mRNA or lentiviral production as follows:

To clone the mSCF CAR, the full-length DNA sequence for secreted murine SCF obtained from UniProt (uniprot.org) (43) was ordered as a gene fragment product flanked by AscI and NheI restriction enzyme sites or BamHI and NotI restriction enzyme sites (Integrated DNA Technologies, Coralville, IA, USA). The gene fragment product was cloned into a recipient plasmid with paired AscI and NheI or BamHI and NotI sites upstream of the CD8α, CD28, and CD3ζ CAR components using corresponding restriction enzymes AscI and NheI or BamHI and NotI (New England BioLabs, Ipswich, MA, USA) behind a UBC or T7 promoter. The DNA construct was codon optimized for expression in T cells by Expression Therapeutics (Tucker, GA, USA) using Expression Cassette Optimization (eCO) technology and confirmed by Sanger sequencing (Genewiz, Burlington, MA, USA).

To clone the hSCF sBite, the coding sequence of secreted isoform of human SCF was obtained from GenBank (M59964.1). The sequence of the SCF signal peptide through mature core fragment was identified as bases 1- 426 of the native cDNA sequence. To generate the sBite, this fragment was combined with a G4S linker and CD3 scFv derived from clone OKT3. The sequence was then codon optimized for expression in γδ T cells by Expression Therapeutics (Tucker, GA, USA) using Expression Cassette Optimization (eCO) technology. The optimized sequence was then synthesized and cloned into a pcDNA3.1-based plasmid using seamless ligation technology by Genscript (Piscataway, NJ, USA) behind a T7 promoter.

### 2.4 Production of mSCF CAR and hSCF sBite mRNA

The mMessage mMachine T7 Transcription kit (Thermo Fisher Scientific, Waltham, MA, USA) was used to produce mSCF CAR mRNA after linearization with restriction enzyme XhoI, whose restriction site cuts directly after the CD3ζ sequence.

The HiScribe T7 mRNA kit with CleanCap Reagent AG Plasmid (New England BioLabs, Ipswich, MA, USA) was used to produce hSCF sBite Cap-1 mRNA after linearization with NotI. Poly(a) tailing was subsequently performed using E. coli poly(a) polymerase (New England BioLabs, Ipswich, MA, USA).

RNA gel electrophoresis was used to confirm efficient poly-A tail extension and quality of mRNA products.

### 2.5 Production of mSCF CAR and CD19 CAR lentiviral vectors

High-titer, recombinant, self-inactivating HIV lentiviral vectors were produced and titered as previously described (44–46). Briefly, HEK-293T cells were transiently transfected with packaging plasmids encoding Gag-Pol, Rev, VSVG envelope, and the CAR transgene plasmid using calcium phosphate (Sigma Aldrich, St. Loius, MO, USA). Cells were cultured in DMEM (Thermo Fisher Scientific, Waltham, MA, USA) supplemented with 10% FBS and supernatant was collected for 3 days beginning 48 hours after transfection. Supernatants were passed through a 0.45-µm filter, pooled, and concentrated by overnight centrifugation at 10,000 × *g* at 4°C. The following morning, viral particles were passed through a 0.22-µm filter and stored at -80°C. Titers were determined using quantitative real-time PCR analysis on HEK-293T cell genomic DNA.

### 2.6 Expansion and modification of primary αβ T cells

Peripheral blood from healthy adult volunteers were obtained under an IRB approved protocol (IRB00101797) through the Emory University Children’s Clinical and Translational Discovery Core (CCTDC), after which peripheral blood mononuclear cells (PBMCs) were isolated using a Ficoll-Plaque PLUS density gradient (GE HealthCare, Chicago, IL, USA) and cryopreserved. Alternatively, cryopreserved PBMCs were procured from AllCells (Alameda, CA, USA). T cells were negatively selected from thawed PBMCs using the EasySep Human T cell Isolation Kit (Stemcell Technologies, Vancouver, BC, CA) and stimulated with anti-CD3/CD28 Human T-Activator Dynabeads (Thermo Fisher Scientific, Waltham, MA, USA) at a 1:1 bead-to-cell ratio in X-VIVO 15 (Lonza, Basel, Switzerland) supplemented with 10% FBS, 1% penicillin-streptomycin, 100 IU/mL human IL-2 (PeproTech, Rocky Hill, NJ, USA), and 5 ng/mL human IL-7 (PeproTech, Rocky Hill, NJ, USA) at 2 × 10^6^ cells/mL. After 48 hours of stimulation, beads were removed and 1 × 10^6^ cells were transduced at MOI 20 with 10 μg/mL polybrene (EMD Millipore, Billerica, MA, USA) for 18 hours. MOI was determined using the following calculation:


$$MOI=\frac{titer\;\times \;volume\;(mL)}{\#\;of\;cells}$$

After 18 hours, cells were resuspended in fresh media and underwent an early cell dilution of ~100,000 cells/mL, then left to expand for 96 hours before CAR expression was determined and used in cytotoxicity assays or animal studies.

### 2.7 Expansion and transfection of primary γδ T cells

Peripheral blood from healthy consenting volunteers (30-40 mL) was obtained as previously mentioned (under the Expansion and modification of primary αβ T cells section) through the CCTDC or fresh leukopaks were procured from the American Red Cross. From either source, PBMCs were isolated using a Ficoll-Plaque PLUS density gradient and cultured in OpTmizer serum-free media (Thermo Fisher Scientific, Waltham, MA, USA) supplemented with 2 mM L-glutamine, 1% penicillin-streptomycin, 500-1000 IU/mL human IL-2, and 5 µM zoledronic acid (Sigma-Aldrich, St. Louis, MO, USA) using our previously published Good Manufacturing Practice-compliant 12-day expansion protocol (31, 33, 35). Expanded γδ T cells were cryopreserved in 5% human serum albumin (HSA; Grifols, Barcelona, Spain) and 10% dimethylsulfoxide (DMSO; Sigma-Aldrich, St. Louis, MO, USA) diluted in PlasmaLyte A (Baxter International Inc. Deerfield, IL, USA) in 1 × 10^7^ – 1 × 10^8^ cell aliquots.

Cryopreserved γδ T cells were thawed in three volumes of 5% HSA diluted in PlasmaLyte A and centrifuged at 250 × *g* for 10 minutes. Cells were resuspended in OpTmizer serum-free media supplemented with 2 mM L-glutamine, 1% penicillin-streptomycin, and 1000 IU/mL of human IL-2 and rested for 2 hours at 37°C, 5% CO_2_. Rested γδ T cells (5-25 × 10^6^ cells) were then transiently transfected to express an mSCF CAR or hSCF sBite through mRNA electroporation by first washing the cells three times with PBS and spinning at 250 × g for 10 minutes, then resuspending in Opti-MEM (ThermoFisher Scientific, Waltham, MA, USA) with 15 μg of purified mRNA or equal volume vehicle control for mock transfection. Cells were then electroporated using a square wave function at 500 V for 5 ms in a 4 mm electroporation cuvette (Fisher Scientific, Hampton, NH, USA) using the GenePulser Xcell Electroporation System (Bio-Rad Laboratories, Hercules, CA, USA). Modified γδ T cells were used 24 hours after electroporation for *in vitro* studies and 1-4 hours after electroporation for *in vivo* studies.

### 2.8 Measuring CAR expression by western blotting

mSCF CAR-modified cells were lysed with RIPA buffer (Sigma-Aldrich, St. Louis, MO, USA) supplemented with Protease Inhibitor Cocktail diluted 1:100 (Sigma-Aldrich, St. Louis, MO, USA) and PMSF protease inhibitor diluted 1:100 (ThermoFisher Scientific, Waltham, MA, USA) for 30 minutes on ice, after which cell lysates were clarified by centrifugation at 14,000 × *g* at 4°C for 10 minutes. The supernatant was collected as a whole cell lysate. Protein concentrate was quantified using a Bradford Assay (Bio-Rad Laboratories, Hercules, CA, USA). 20-40 μg of protein was first heated at 100°C for 10 minutes, then loaded into a 4-15% pre-cast gel (Bio-Rad Laboratories, Hercules, CA, USA) with Precision Plus Protein Dual Color Standards (Bio-Rad Laboratories, Hercules, CA, USA) and separated by SDS-PAGE. Protein was transferred to a nitrocellulose membrane (Bio-Rad Laboratories, Hercules, CA, USA) at 250 constant Amps for 60 minutes and then washed in TBS containing 0.1% Tween-20 prior to incubation with a mouse anti-human CD3ζ primary antibody diluted 1:1000 (BioLegend, San Diego, CA, USA) and goat anti-mouse HRP secondary antibody diluted 1:500 (BioLegend, San Diego, CA, USA). Blots were then imaged by chemiluminescence with a Bio-Rad ChemiDoc XR+ Molecular Imager.

### 2.9 Measuring CAR expression by flow cytometry

Jurkat or primary CAR T cells were stained with 20 ng of Recombinant Mouse c-kit Fc Chimera Protein (R&D Systems, Minneapolis, MN, USA) or 20 ng of Recombinant Human c-kit Fc Chimera Protein (R&D Systems, Minneapolis, MN, USA) and incubated on ice for 30 minutes. Cells were washed once with FACS buffer (PBS + 2.5% FBS), then stained with 2 μL R-Phycoerythrin F(ab’)2 secondary antibody (Jackson ImmunoResearch, West Grove, PA, USA) and eBioscience Fixable Viability Dye eFluor 780 (Thermo Fisher Scientific, Waltham, MA, USA) to exclude dead cells and incubated on ice for 15 minutes. Cells were then washed once with FACS buffer, then subjected to flow cytometry (Cytek Aurora) and analyzed with FlowJo software (v10).

### 2.10 *In vitro* cytotoxicity assays

To assess the cytotoxicity of mSCF CAR- or hSCF sBite-T cells against AML cell lines, unmodified or modified primary T cells were co-cultured with 50,000 target cells (CMK or Kasumi-1) stained with VPD450 (BD, Franklin Lakes, NJ, USA) at indicated effector-to-target ratios for 4-24 hours at 37°C, 5% CO_2_. Co-cultures were then washed once with Annexin V Binding Buffer (0.025 mM calcium chloride + 1.4 mM sodium chloride + 0.1 mM HEPES) and stained with 3 μL Annexin V-APC (BioLegend, San Diego, CA, USA) for 20 minutes at room temperature. Cells were then washed once with Annexin V Binding Buffer. 7-AAD Viability Dye (BioLegend, San Diego, CA, USA) was added 2 minutes before the sample was subjected to flow cytometric analysis (Cytek Aurora). Samples were analyzed with FlowJo software (v10), where percent cytotoxicity was measured by the sum of Annexin V^+^, 7-AAD^+^, and Annexin-V^+^/7-AAD^+^ target cells.

### 2.11 *In vitro* activation assays

Jurkat or primary CAR T cells were co-cultured with VPD450 (BD, Franklin Lakes, NJ, USA) stained target cells (CMK, Kasumi-1, Nomo-1, or 697) at indicated effector-to-target ratios for 4 hours at 37°C, 5% CO_2_. Co-cultures were then washed once with FACS buffer and stained with 3 μL anti-CD69-APC-Cy7 antibody (BD, Franklin Lakes, NJ, USA) for 20 minutes at 4°C. After staining, cells were washed once with FACS buffer, then subjected to flow cytometry (Cytek Aurora) and analyzed with FlowJo software (v10). Dead cells were excluded from the analysis using 3 μL of 7-AAD Viability Dye added 2 minutes before analysis on the cytometer.

### 2.12 Colony forming unit assay

For each bone marrow sample, 0.8-8 × 10^4^ cells isolated from mouse femurs by flushing were first resuspended in RPMI 1640 supplemented with 10% FBS and 1% penicillin-streptomycin. Resuspended cells were then added to 4 mL Methocult GF M3434 (Stemcell Technologies, Vancouver, BC, CA) and plated on sterile 35 mm plates in triplicate. Plates were incubated at 37°C 5% CO_2_ for 7 days, then colonies were counted under a microscope.

### 2.13 Animal studies

NOD.Cg-Prkdc^scid^Il2rg^tw1Wjl^/SzJ (NSG) mice were purchased from Jackson Laboratories (Bar Harbor, ME, USA) and maintained in a pathogen-free environment at an Emory University Division of Animal Resources facility. All animal studies were conducted in accordance with established policies set forth by the Emory University Institutional Animal Care and Use Committee (IACUC) under an approved animal use protocol (PROTO201800202). Equal numbers of male and female mice were used for all studies.

To determine the toxicity of mSCF CAR-modified αβ T cells, 8-week-old NSG mice were first preconditioned with 100 rads of x-ray irradiation (Rad Source Technologies, RS 2000 Small Animal Irradiator, Buford, GA, USA) then administered 5 × 10^6^ luciferase-modified CMK cells by intravenous injection into the lateral tail-vein the following morning. The following day, mice were either treated with 5 × 10^6^ mock-transduced αβ T cells (n = 6) or mSCF CAR-modified αβ T cells (n = 6, transduction efficiency:<8%) via tail-vein injection, with PBS administered as an untreated control (n = 3). Peripheral blood was collected at 3 weeks to assess for tumor burden and CAR T-cell engraftment. Mice were euthanized when end-point criteria were met, which includes changes in weight, scruff, movement, and hunched state.

To examine the persistence of γδ T cells in mice, 7-13-week-old NSG mice were administered 1 × 10^7^ non-modified γδ T cells via retro-orbital injection. In an effort to enhance persistence, mice were either treated with vehicle control (n = 6), 13,000 IU IL-2 twice a week via intraperitoneal injection (n = 6), or a combination of 13,000 IU IL-2 twice a week via intraperitoneal injection and 70 μg/kg zoledronic acid (NorthStar Rx, Memphis, TN, USA) via subcutaneous injection (n = 6). Once a day for 4 days, peripheral blood was collected, and leukocytes were assessed for the presence of human CD45^+^ cells by flow cytometric analysis (Cytek Aurora) and analyzed with FlowJo software (v10). On day 4, mice were euthanized, and spleens and bone marrow were harvested to assess for the presence of human CD45^+^ cells as before.

To examine the toxicity of mSCF CAR-modified γδ T cells in mice, 8-12-week-old NSG mice were anesthetized and administered 2-8 × 10^6^ mock transfected γδ T cells (n = 4) or mSCF CAR-modified γδ T cells (n = 4), with PBS as an untreated control (n = 3) via intraosseous injection in the left femur. Mice were given 5 mg/kg meloxicam (Baudax Bio, Malvern, PA, USA) via subcutaneous injection immediately after the intraosseous injection as an analgesic. After 2 days, mice were euthanized and peripheral blood, spleens, and bone marrow from both the left and the right femur were harvested to assess for the presence of human CD3^+^ human γδTCR^+^ cells and murine c-kit^+^ cells by flow cytometry (Cytek Aurora). Data was analyzed using the FlowJo software (v10). At this time, a CFU assay was performed to assess for multipotent progenitor cells. The left and the right femurs from each mouse were kept as separate samples in the flow and CFU assays in this experiment.

To assess the cytotoxicity of mSCF CAR- and hSCF sBite-modified γδ T cells in an AML model, 5–9-week-old NSG mice were first preconditioned with 20 mg/kg busulfan (DSM Pharmaceuticals, Inc., Greenville, NC, USA) via intraperitoneal injection, then administered 5 × 10^6^ luciferase-modified CMK cells via tail-vein injection the following morning. Beginning in the afternoon on the same day and then once daily for the following 3 days for a total of 4 doses, mice were treated with 1 × 10^7^ mock transfected γδ T cells (n = 6), mSCF CAR-modified γδ T cells (n = 3), or hSCF sBite-modified γδ T cells (n = 6) by intravenous injection into the lateral tail-vein, with PBS to serve as an untreated control (n = 8). Tumor growth and overall health of the mice were monitored two times per week IVIS (*In vivo* Imaging System, Perkin Elmer, Waltham, MA, USA) imaging and weighing, respectively. Freshly made D-luciferin (PerkinElmer, Waltham, MA, USA) was injected at 150 mg/kg via intraperitoneal injection 10 minutes prior to imaging. Bioluminescence was quantified using Living Image Software (PerkinElmer, Waltham, MA, USA) or Aura Software (Spectral Instruments Imaging, Tucson, AZ, USA). Peripheral blood was collected at 3 weeks to assess for c-kit expression on CD33^+^ AML cells and to perform complete blood counts. Mice were euthanized when end-point criteria were met, which includes changes in weight, scruff, movement, and hunched state.

### 2.14 Statistical analysis

All statistical analyses were performed using Prism 8 software (GraphPad Software Inc). Results are presented as mean ± standard deviation of the mean and were considered statistically significant at *P*< 0.05. Unpaired or paired two-tailed Student’s *t*-test, one-way ANOVA, or log-rank (Mantel-Cox) test were used to determine statistical significance as appropriate.

## 3 Results

### 3.1 c-kit is highly expressed on pediatric AML and healthy HSCs

**Figure 1:**
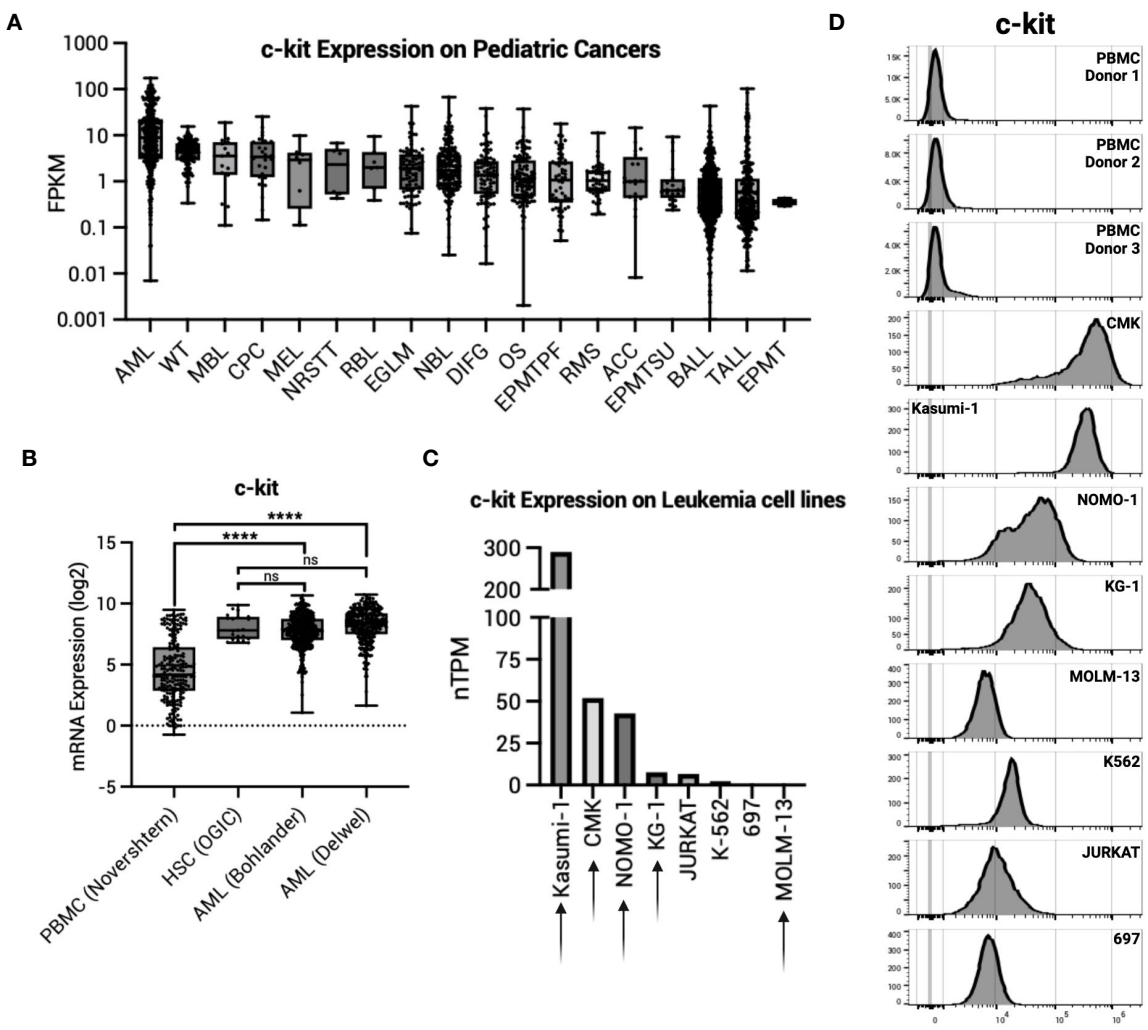
c-kit expression on AML. **(A)** c-kit expression as fragments per kilobase per million (FPKM) on pediatric cancers from the St. Jude Cloud (pecan.stjude.cloud). Error bars represent SD. **(B)** c-kit mRNA expression across one normal PBMC dataset, one normal HSC dataset, and two AML datasets from R2: Genomics Analysis and Visualization Platform (https://r2.amc.nl). Error bars represent SD. Statistical analysis represents One-Way ANOVA (****p< 0.0001; ns, p > 0.05). **(C)** c-kit expression as nTPM on select leukemia cell lines from the Human Protein Atlas (v22.proteinatlas.org). Arrow denotes AML cell line. **(D)** Histograms depicting c-kit expression in healthy donor PBMCs (n = 3) and select leukemia cell lines in **(C)**.

We first sought to determine c-kit expression on healthy tissues. The Human Protein Atlas (proteinatlas.org) demonstrates undetectable c-kit expression in most adult human organs by immunohistochemistry (IHC). Exceptions to this include high protein expression in the cerebellum, medium protein expression in the female reproductive organs (breast, fallopian tubes, and ovary), lung, skin, and testis, and low protein expression in the kidney, colon, and rectum. Data from the St. Jude Cloud (pecan.stjude.cloud) show many pediatric malignancies have high c-kit expression, with AML as the highest (**Figure 1A**). High expression of c-kit is corroborated in adult AML samples taken at time of diagnosis, with expression significantly higher than normal PBMCs yet comparable to HSCs (**Figure 1B**). To validate these findings, we confirmed c-kit expression on eight specific leukemia cell lines (proteinatlas.org) (**Figure 1C**) and by flow cytometry (**Figure 1D**). Four of five AML cell lines tested (AML cell lines denoted by arrows in **Figure 1C**) demonstrate high c-kit expression compared to three healthy donor PBMCs (**Figure 1D**).

### 3.2 mSCF CAR αβ T cells induce AML cell death *in vitro*

**Figure 2:**
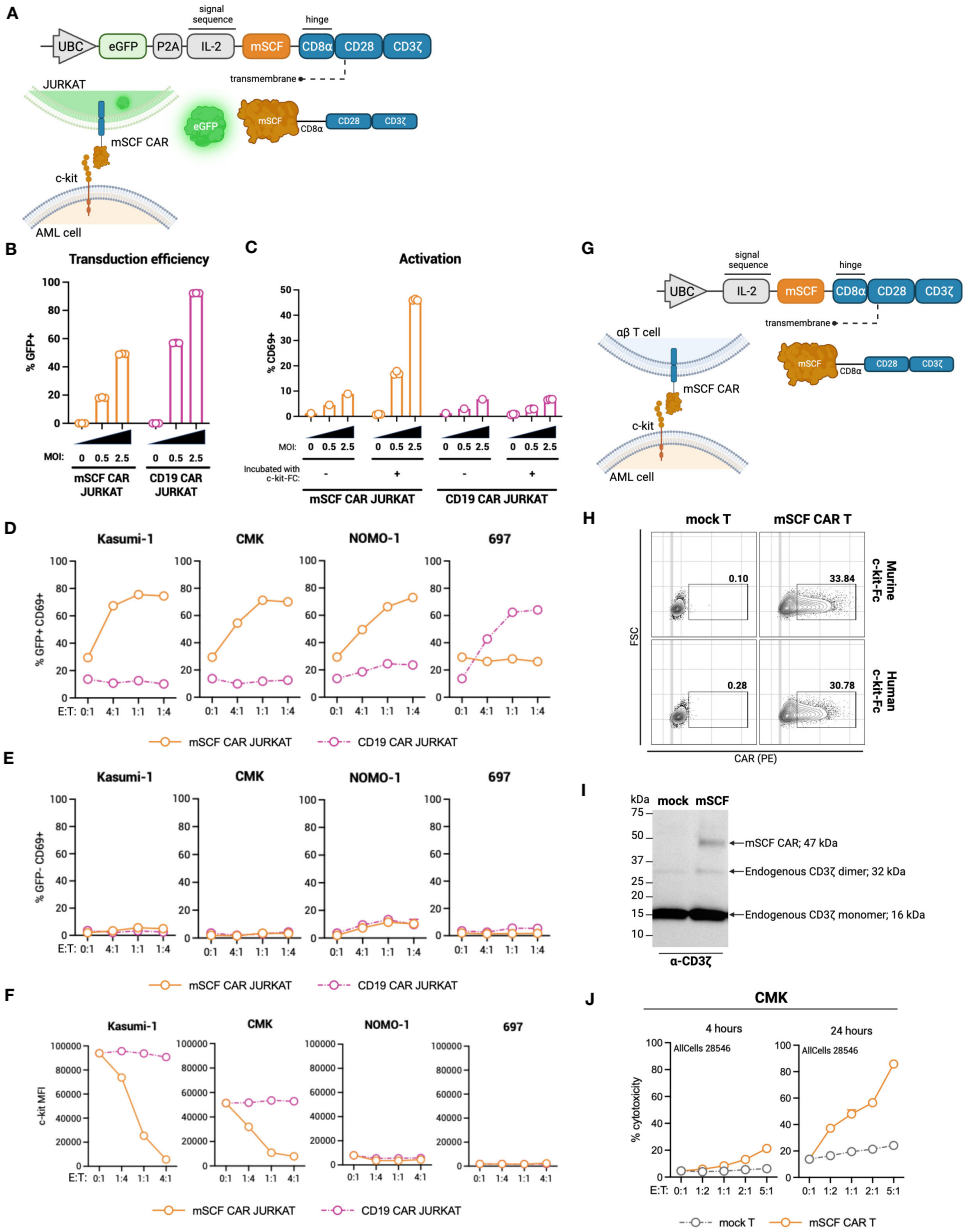
Design of novel ligand-based mSCF CAR and modification of αβ T cells. **(A)** Schematic of lentiviral GFP mSCF CAR DNA construct. **(B)** Transduction efficiency as depicted by %GFP^+^ live Jurkat T cells. Error bars represent SD. n = 3 experimental replicates. **(C)** Activation of live mSCF CAR- or CD19 CAR-modified Jurkat T cells as depicted by %CD69^+^ when stained with 20 ng murine c-kit-Fc chimera. Error bars represent SD. n = 1-3 experimental replicates. **(D)** %CD69 activation of live mSCF CAR- or CD19 CAR-modified GFP^+^ Jurkat T cells when co-cultured with 4 different leukemia cell lines at 4 different effector-to-target (E:T) ratios. Error bars represent SD. n = 2 experimental replicates. **(E)** %CD69 activation of live un-modified GFP- Jurkat T cells under the same conditions as **(D)**. Error bars represent SD. n = 2 experimental replicates. **(F)** c-kit MFI of 4 leukemia cell lines when co-cultured with mSCF CAR- or CD19 CAR-modified Jurkat T cells under the same conditions as **(D)**. Error bars represent SD. n = 2 experimental replicates. **(G)** Schematic of lentiviral mSCF CAR DNA construct without GFP marker. **(H)** Representative flow plots depicting mSCF CAR expression on primary T cells transduced at MOI 20 when stained with 20 ng of murine or human c-kit-Fc chimera. **(I)** Western blot depicting CAR expression from whole cell lysates of primary T cells transduced with mSCF CAR at MOI 20. Western blot antibody against human CD3ζ. **(J)** Four- and 24-hour flow cytometry cytotoxicity assays of mSCF CAR-modified primary T cells against c-kit expressing AML cell line CMK compared to mock T cell controls. Percent cytotoxicity is the sum of 7AAD^+^, Annexin V^+^, and 7AAD^+^ Annexin V^+^ cells when gated on VPD450-stained target cells only. Error bars represent SD. n = 2 experimental replicates with 1 donor.

To this end, we generated a ligand-based c-kit directed CAR (mSCF CAR) by lentiviral gene delivery. The CAR construct includes full-length murine SCF followed by a CD8α hinge, CD28 co-stimulatory domain, and CD3ζ signaling domain (**Figure 2A**). We chose to use the murine sequence for SCF as it binds both the human and murine c-kit receptor (47), making it possible to evaluate both cancer eradication and off-tumor toxicities in a murine model.

The first transgene tested included a bicistronic lentiviral vector encoding for dual expression of eGFP by a P2A ribosomal skipping sequence as a transduction marker (**Figure 2A**). Binding of the murine c-kit receptor to the mSCF CAR can be determined using a murine c-kit receptor conjugated to a constant fragment (Fc; c-kit-Fc) (**Figure S1A**). Jurkat T cells were used to evaluate CAR protein expression, ability to bind the human c-kit receptor, and AML specificity, with antigen-irrelevant CD19 CAR as a control. Jurkat T cells transduced with mSCF CAR at multiplicities of infection (MOIs) 0.5 and 2.5 resulted in 18% ± 0.4% and 49% ± 0.3% GFP^+^ cells respectively, while cells transduced with the control CD19 CAR resulted in 57% ± 0.2% and 92% ± 0.1% GFP^+^ cells, respectively (**Figure 2B**). Importantly, binding of the c-kit-Fc to mSCF CAR-modified Jurkat T cells results in CD69 upregulation and subsequent activation increasing with increasing MOIs, but not in CD19 CAR-modified Jurkat T cells (**Figure 2C**). Interestingly, this increase in activation is only seen when both c-kit-Fc and secondary F(ab’)2 are used (**Figure S2B**), suggesting multiple binding domains of the secondary F(ab’)2 may potentially induce CD3ζ cross-linking among CAR molecules (**Figure S1C**), though further evidence is required.

To assess interaction of the mSCF CAR with human c-kit and the ability to activate Jurkat T cells, mSCF CAR Jurkat T cells were co-cultured with c-kit^+^ human cell lines Kasumi-1, CMK, and Nomo-1 at various effector-to-target (E:T) ratios, with the B-cell leukemia cell line 697 as an antigen-negative control, as it does not express c-kit. CD19 CAR Jurkat T cells were used as an antigen-irrelevant control for co-cultures with c-kit^+^ cell lines, as they do not express CD19, and a positive control when co-cultured with 697 cells, as 697 cells do express CD19. Indeed, transduced (GFP^+^) Jurkat T cells resulted in increased CD69^+^ activation when co-cultured with their antigen-matched target cells (**Figure 2D**), while un-transduced (GFP^-^) Jurkat T cells do not exhibit increased CD69^+^ activation (**Figure 2E**). Furthermore, it is known that interaction of SCF with the c-kit receptor causes receptor internalization (48). Indeed, co-culture of mSCF CAR Jurkat T cells with c-kit^+^ cell lines resulted in decreased c-kit MFI, while MFI remain unchanged when target cells were co-cultured with CD19 CAR Jurkat T cells (**Figure 2F**).

As a ligand-based therapeutic, it is important to consider the consequences of CAR shedding, as additional SCF in circulation may promote cancer cell proliferation (48). To assess CAR shedding, c-kit^+^ AML cell lines CMK and Kasumi-1 were incubated with media from mSCF CAR αβ T cells or CD19 CAR αβ T cells, with mock αβ T cells as a control. As a positive control, cell lines were incubated with T cell media supplemented with recombinant murine SCF to induce receptor internalization. After a 15-minute incubation, there is no evidence of receptor internalization when cells were incubated with mSCF CAR media, suggesting the mSCF CAR is not shed from the surface of the cell at levels sufficient to influence surface c-kit levels (**Figure S2**).

To investigate the mSCF CAR in a more clinically relevant setting, we tested a second lentiviral mSCF CAR transgene cassette that had eGFP removed in primary αβ T cells (**Figure 2G**). Primary αβ T cells were transduced at MOI 20, after which binding of mSCF CAR αβ T cells to both murine and human c-kit-Fc was confirmed by flow cytometry (**Figure 2H**) and overall protein expression was confirmed by western blotting against CD3ζ (**Figure 2I**). To assess cytotoxicity, mSCF CAR αβ T cells were co-cultured with c-kit^+^ CMK cells at increasing E:T ratios for 4- or 24-hours and showed increased cytotoxicity compared to mock αβ T controls (**Figure 2J**). Specifically, after 24-hours of co-culture, mSCF CAR αβ T cells induced cytotoxicity to an average of 37% at a 1:2, 48% at a 1:1, 57% at a 2:1, and 86% at a 5:1 E:T ratio. Together, these data show mSCF CAR T cells induce efficient and c-kit-specific killing of c-kit^+^ AML cell lines *in vitro*.

### 3.3 mSCF CAR αβ T cells expand *in vivo* in the absence of AML

**Figure 3:**
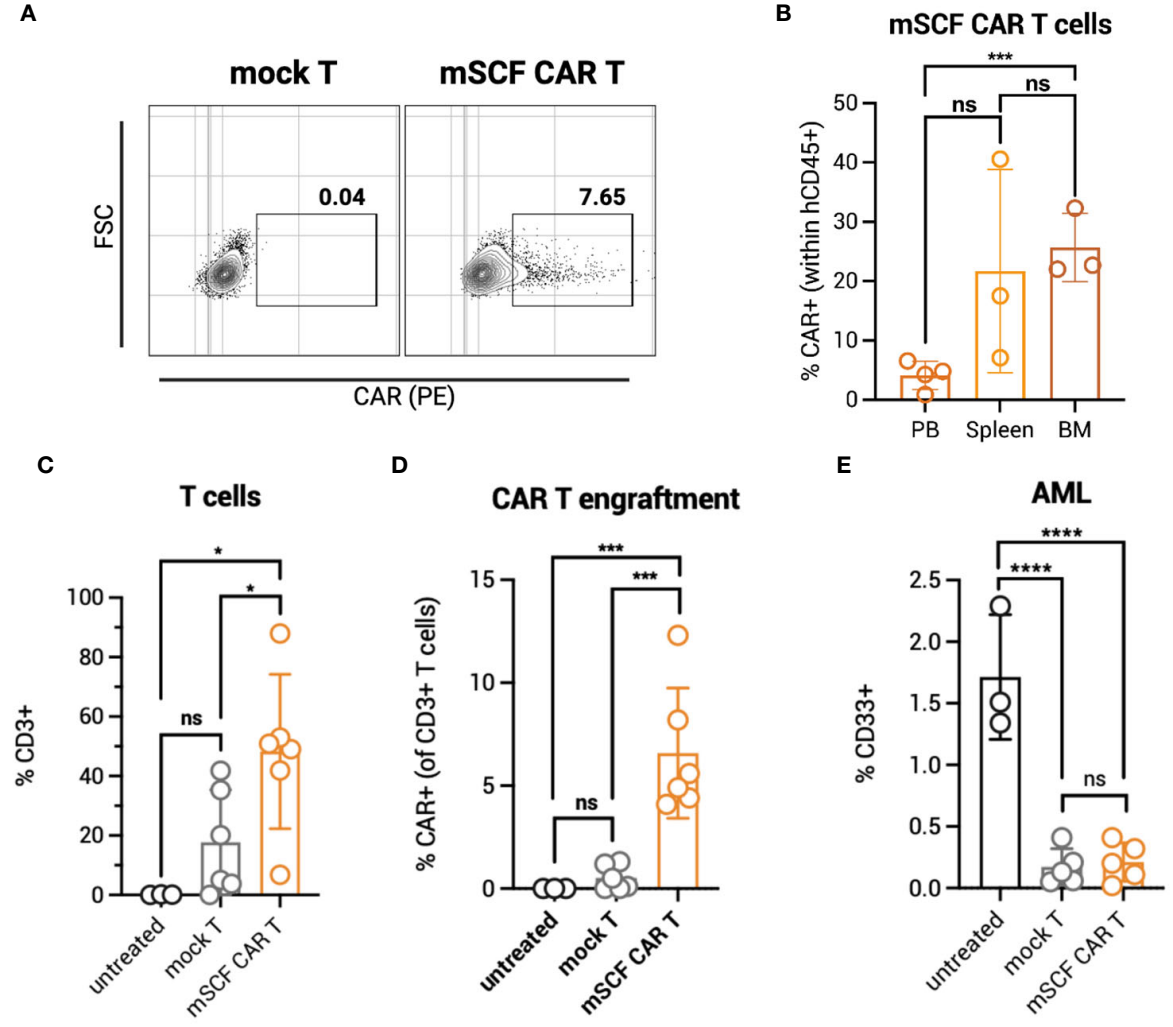
mSCF CAR αβ T cells expand in vivo in the absence of AML. **(A)** Representative flow plots depict mSCF CAR expression of injected αβ T cells in the naive context and in the context of AML. **(B)** Briefly, NSG mice were injected with 5 x 10^6^ mSCF CAR-modified αβ T cells intraveneously in the absence of AML. %CAR+ αβ T cells (gated on live human CD45+ cells) within the peripheral blood (PB), spleen, and bone marrow (BM) compartments. Error bars represent SD. Statistical analysis represents Student's t test (***p<0.001; ns, p > 0.05). **(C)** Briefly, NSG mice recieved 100 rads of x-ray irradiation in the morning followed by IV injection of 5 x 10^6^ CMK cells in the afternoon on day -1. The following day, mice were treated with 5 x 10^6^ mock αβ T cells or mSCF CAR-modified αβ T cells (<8% CAR+) intraveneously. Peripheral blood leukocytes were collected 3 weeks after treatment to assess for leukemia engraftment. n = 3 untreated, n = 6 mock T, n = 6 mSCF CAR T. %CD3 live αβ T cells circulating within the periphery 3 weeks after treatment. Error bars represent SD. Statistical analysis represents Student's t test (*p<0.05; ns, p > 0.05). **(D)** %CAR+ αβ T cells (gated on live human CD3+ cells) circulating within the periphery 3 weeks after treatment. Error bars represent SD. Statistical analysis represents Student’s t test (***p<0.001; ns, p > 0.05). **(E)** %CD33+ live AML cells circulating within the periphery 3 weeks after treatment. Error bars represent SD. Statistical analysis represents Student's t test (****p < 0.0001; ns, p > 0.05).

To identify possible toxicities of mSCF CAR αβ T cells *in vivo*, NOD.Cg-Prkdc^scid^Il2rg^tw1Wjl^/SzJ (NSG) mice were administered 5 × 10^6^ total mSCF CAR-modified αβ T cells by tail-vein injection. To assess clonal expansion of the CAR^+^ population *in vivo*, αβ T cells were transduced at low MOIs, providing approximately 8% CAR^+^ cells (**Figure 3A**). Six weeks after administration of T cells, mice were sacrificed to assess clonal expansion of CAR^+^ cells in the peripheral blood, spleen, and bone marrow compartments by flow cytometry. Significantly more CAR^+^ αβ T cells were found in the bone marrow (26% ± 6%) than in the peripheral blood (5% ± 1%) (**Figure 3B**), with the percentage of CAR^+^ cells found in the peripheral blood closely resembling percent CAR^+^ cells injected at the beginning of the study.

To test clonal expansion in the presence of AML cells, NSG mice were first subjected to 100 rads of x-ray irradiation, then inoculated with 5 × 10^6^ luciferase-modified CMK cells by tail-vein injection. The following day, mice were intravenously injected with one dose of 5 × 10^6^ total mock or mSCF CAR αβ T cells (8% CAR^+^). Three weeks later, circulating human CD3^+^ T cells were significantly higher in mSCF CAR αβ T-cell treated mice (48% ± 26%) compared to mock αβ T-cell treated mice (18% ± 18%) (**Figure 3C**). No expansion of CAR^+^ cells was observed in the periphery, as only 7% ± 3% CAR^+^ cells were found in circulation three-weeks after injection, resembling the percentage of CAR^+^ cells in the injected product (**Figure 3D**).

CD33^+^ AML burden in the periphery was significantly decreased in mSCF CAR-treated mice (0.2% ± 0.2%) compared to untreated controls (2% ± 0.5%) three-weeks after treatment, though not significantly different than mock αβ T-cell treated mice (0.2% ± 0.2%) (**Figure 3E**). However, despite a lack of difference in AML burden in the periphery between treatment groups, mSCF CAR αβ T cell treated mice met endpoint criteria significantly sooner than untreated or mock αβ T treated mice, suggesting greater toxicity *in vivo* despite low CAR transduction (**Figure S3**).

Though we did not observe clonal expansion of CAR^+^ αβ T cells in the periphery of AML bearing mice, we hypothesized there could be clonal expansion in other hematopoietic organs with high c-kit expression, such as the bone marrow. Despite promising *in vitro* data, we determined that mSCF CAR-modified αβ T cells induced excessive toxicity *in vivo* even at low modification efficiency. Thus, we sought additional safety modifications in our design.

### 3.4 c-kit-targeting γδ T cells induce AML cell death *in vitro*

**Figure 4:**
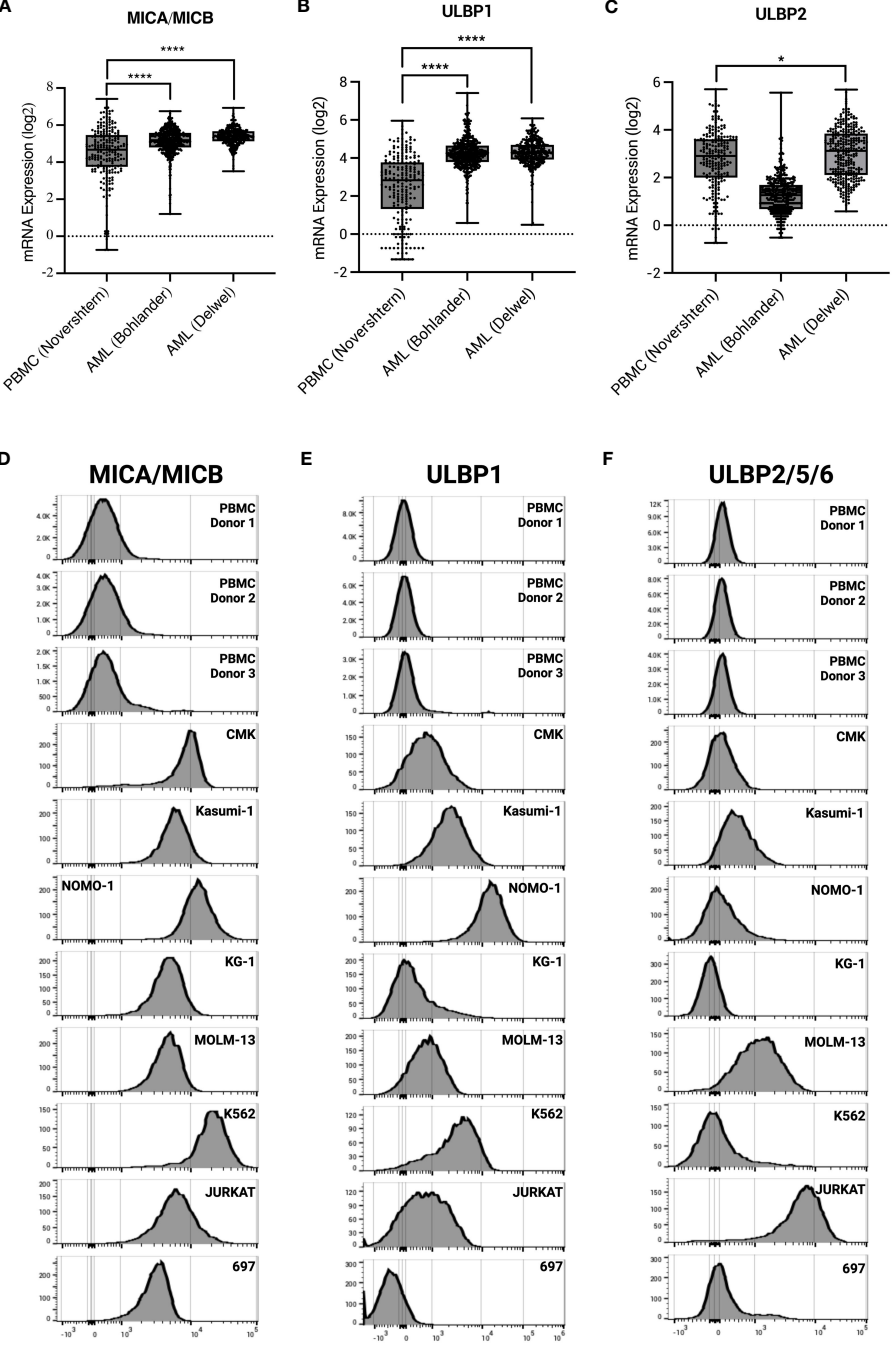
AML and leukemia cell lines express sress antigens MICA/MICB, ULBP1, and ULBP2/5/6. mRNA expression of stress antigens MICA/MICB **(A)**, ULBP1 **(B)**, and ULBP2 **(C)** on normal PBMC and two AML datasets queried using R2: Genomics Analysis and Visualization Platform (https://r2.amc.nl). Error bars represent SD. Statistical analysis represents One-Way ANOVA (****p < 0.0001; *p < 0.05). Histograms depict stress antigen expression of MICA/MICB **(D)**, ULBP1 **(E)**, and ULBP2/5/6 **(F)** in healthy donor PBMC (n = 3) and leukemia cell lines by flow cytometry.

γδ T cells have an innate ability to recognize stress antigens through NKG2D expression (49). NKG2D ligands—MICA/MICB and ULBP1-6—interact with NKG2D on γδ T cells and activate innate killing mechanisms (50). Primary AML samples taken at time of diagnosis from two different datasets express significantly higher transcript levels of NKG2D ligands MICA/MICB, ULBP1, and ULBP2 compared to healthy PBMC control (**Figures 4A–C**). These results were corroborated with cell surface expression on AML cell lines as measured by flow cytometry (**Figures 4D–F**).

**Figure 5:**
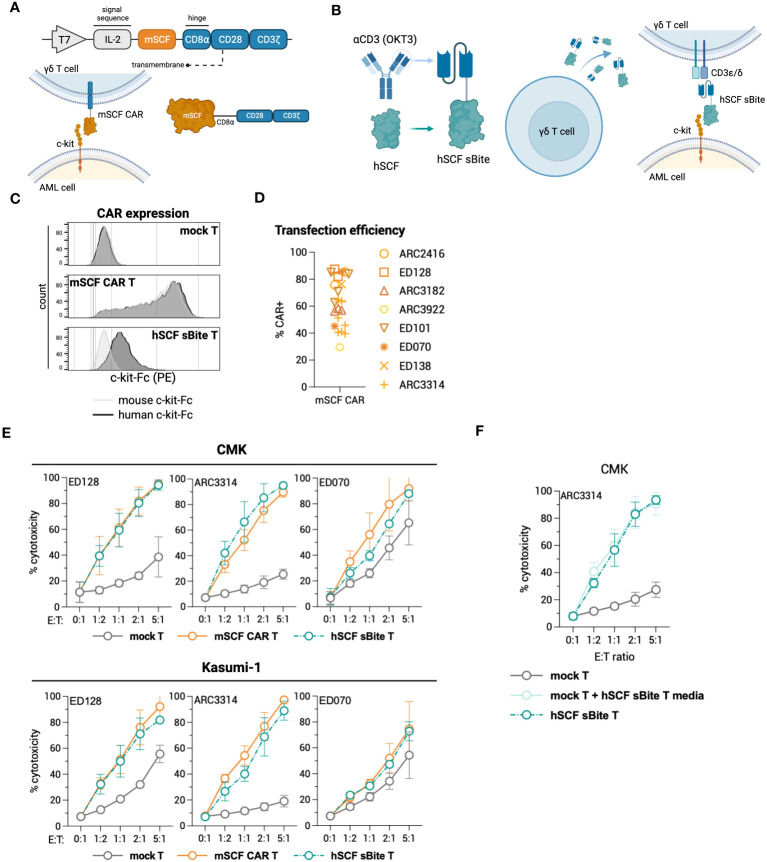
Design of novel ligand-based therapeutics and transient modification of γδ T cells. **(A)** Schematic of mSCF CAR construct for mRNA generation. **(B)** Diagram of hSCF sBite construct for mRNA generation. **(C)** Representative flow plots depicting mSCF CAR expression on primary γδ T cells transfected with 15 μg mRNA encoding denoted construct when stained with 20 ng of murine or human c-kit-Fc chimera. **(D)** Pooled transfection efficiency of primary γδ T cells modified with the mSCF CAR. n = 8 donors with n = 1-7 biological replicates. **(E)** Four-hour flow cytometry cytotoxicity assays of mSCF CAR- and hSCF sBite-modified primary γδ T cells against c-kit expressing AML cell lines compared to mock γδ T cell controls. Percent cytotoxicity is the sum of 7AAD^+^, Annexin V^+^, and 7AAD^+^ Annexin V^+^ cells when gated on VPD450-stained target cells only. Error bars represent SD. n = 3 donors with n = 2-7 biological replicates each. **(F)** Four-hour flow cytometry cytotoxicity assay of hSCF sBite γδ T cells against c-kit^+^ CMK cells compared to mock γδ T cell control. Mock γδ T cells were co-cultured with media from hSCF sBite-modified cells and c-kit^+^ CMK cells to measure sBite secretion. Error bars represent SD. n = 1 donor with n = 4 biological replicates.

To test c-kit-directed ligand-based therapeutics in γδ T cells, we developed a transgene to express the mSCF CAR under a T7 promoter for mRNA production (**Figure 5A**). Similarly, we also developed a ligand-based secreted bispecific T-cell engager (sBite) using the human sequence for SCF and an anti-CD3 scFv (clone: OKT3) (**Figure 5B**). When expressed in a γδ T cell, the hSCF sBite is secreted and dually binds to the γδ T-cell receptor (TCR) and c-kit, which is expressed on the target cell, and induces cytotoxicity. As γδ T cells have innate cytotoxicity against cancer cells, we posited that expressing these targeted transgenes transiently would provide potent tumor clearance without long-term off-target toxicities within the c-kit compartment. Flow cytometric analysis confirms CAR surface expression using mouse and human c-kit-Fc (**Figure 5C**). Interestingly, γδ T cells modified with the hSCF sBite shift positively when incubated with the human c-kit-Fc, suggesting sBite secretion, binding to surface CD3ϵ and ability to bind to human c-kit. Importantly, this also confirms that the human sequence for SCF is unable to bind to murine c-kit (**Figure 5C**). Overall, mSCF CAR transfection efficiency averages to 63% ± 18% from samples across eight healthy donors (**Figure 5D**).

To assess cytotoxicity of mSCF CAR and hSCF sBite-modified γδ T cells, human AML cell lines CMK and Kasumi-1 were co-cultured for 4-hours with mock γδ T cells, mSCF CAR γδ T cells, or hSCF sBite γδ T cells at increasing E:T ratios (**Figure 5E**). As expected, the degree of toxicity of non-modified γδ T cells against some AML cell lines is donor dependent (35). Cytotoxicity increased as E:T ratios increased across the three donors to variable degrees and importantly, CAR and sBite modification enhanced cytotoxicity in all donors (**Figure 5E**). To further confirm secretion of the hSCF sBite from modified γδ T cells, c-kit^+^ CMK cells were co-cultured with mock γδ T cells, mock γδ T cells supplemented with hSCF sBite conditioned media, or hSCF sBite-modified γδ T cells at increasing E:T ratios. Mock γδ T cells supplemented with hSCF sBite conditioned media induced the same degree of cytotoxicity as hSCF sBite-modified γδ T cells, indicating the hSCF sBite is secreted into the media and can act on both modified and non-modified cells (**Figure 5F**).

### 3.5 c-kit-targeting mSCF CAR γδ T cells induce sca-1^+^ cell death *ex vivo*

While hSCF sBite-modified γδ T cells should not induce toxicity against murine c-kit^+^ cells in the bone marrow, as human SCF does not bind to murine c-kit, it is still important to consider the toxicity of mSCF CAR-modified γδ T cells against murine c-kit^+^ cells in the bone marrow. To accomplish this, mSCF CAR γδ T cells and hSCF sBite γδ T cells were co-cultured for 24-hours with sca-1^+^ isolated murine bone marrow at an E:T ratio of 1:2. Importantly, cells were co-cultured in both the absence and presence of supraphysiological levels of recombinant murine SCF to assess the functionality of a ligand-based therapy in a ligand competing environment.

**Figure 6:**
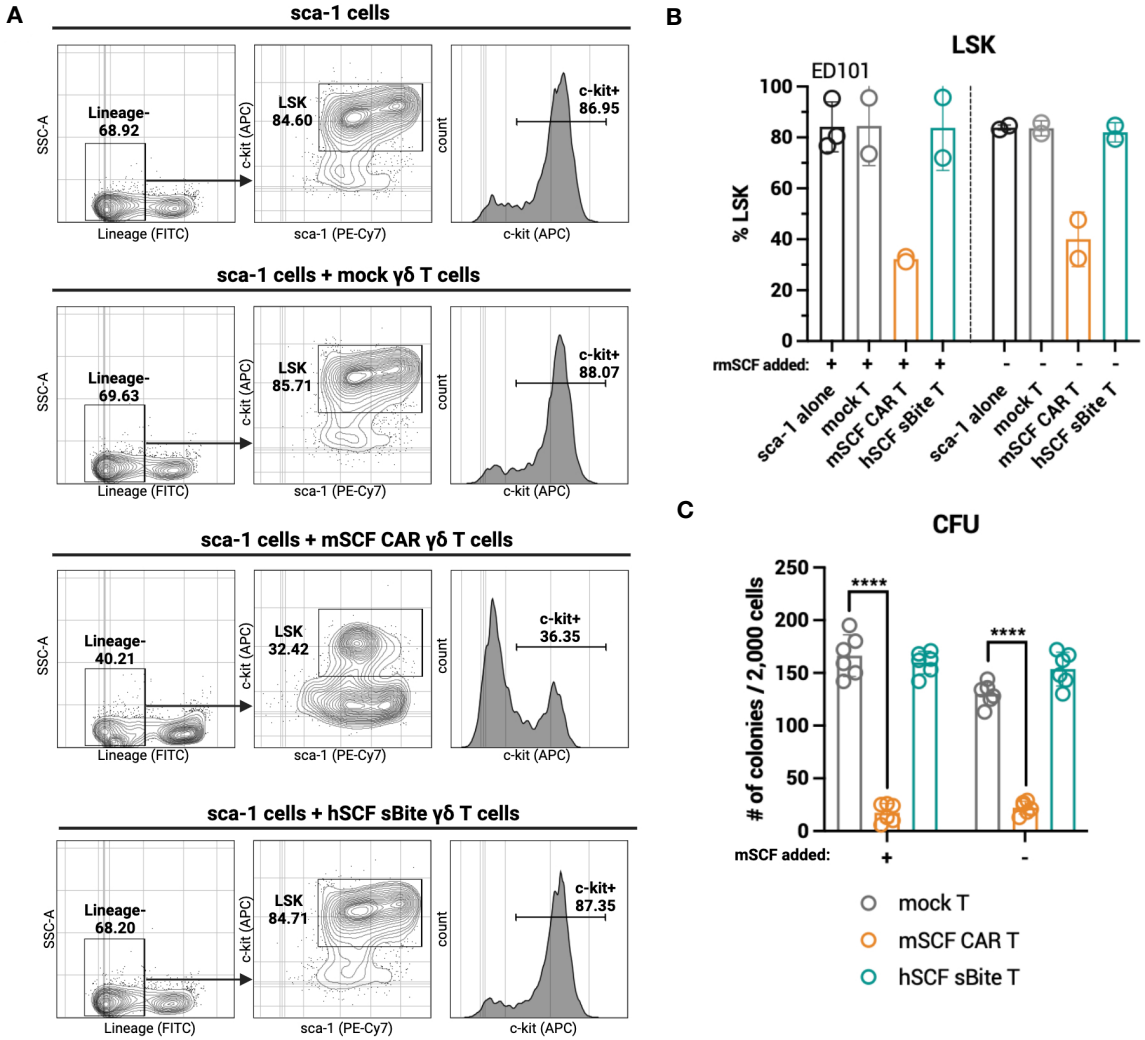
mSCF CAR-modified γδ T cells, but not hSCF sBite-modified γδ T cells, are cytotoxic against murine bone marrow *ex vivo*. **(A-C)** Briefly, murine sca-1^+^ cells were harvested from bone marrow of C57BL/6 mice, rested for 1 day in media supplemented with mIL-3 (20 ng/mL), hIL-11 (100 ng/mL), and hFlt3 (100 ng/mL) and with or without mSCF (100 ng/mL), then co-cultured with γδ T cells for 24-hours at a 1:2 E:T ratio. Co-cultures were subject to flow cytometry analysis to assess LSK and c-kit^+^ compartments. **(A)** Representative flow plots depict LSK and c-kit^+^ compartments of murine sca-1^+^ cells in a 24-hour *ex vivo* co-culture of mock γδ T cells, mSCF CAR γδ T cells, or hSCF sBite γδ T cells supplemented with all cytokine excluding mSCF. **(B)** %LSK (gated on live hCD3^-^ hγδTCR^-^ cells). Error bars represent SD. n = 2-3 biological replicates. **(C)** Number of colonies counted from colony forming unit (CFU) assay after 24-hour co-culture. Error bars represent SD. n = 3 technical replicates. n = 2 biological replicates.

After 24-hours, mSCF CAR γδ T cells, but not hSCF sBite or mock γδ T cells, reduced the c-kit^+^ and Lineage^-^ sca-1^+^ c-kit^+^ (LSK) compartments of murine bone marrow (**Figures 6A, B**). A colony forming unit (CFU) assay cultured at a 1:2 E:T ratio confirms a depletion of progenitor cells in only the cells co-cultured with mSCF CAR γδ T cells (**Figure 6C**). Importantly, this depletion can be seen in both the presence and absence of recombinant murine SCF, suggesting ligand competition inhibiting functionality is minimal.

### 3.6 γδ T cells have limited persistence in NSG mice

**Figure 7:**
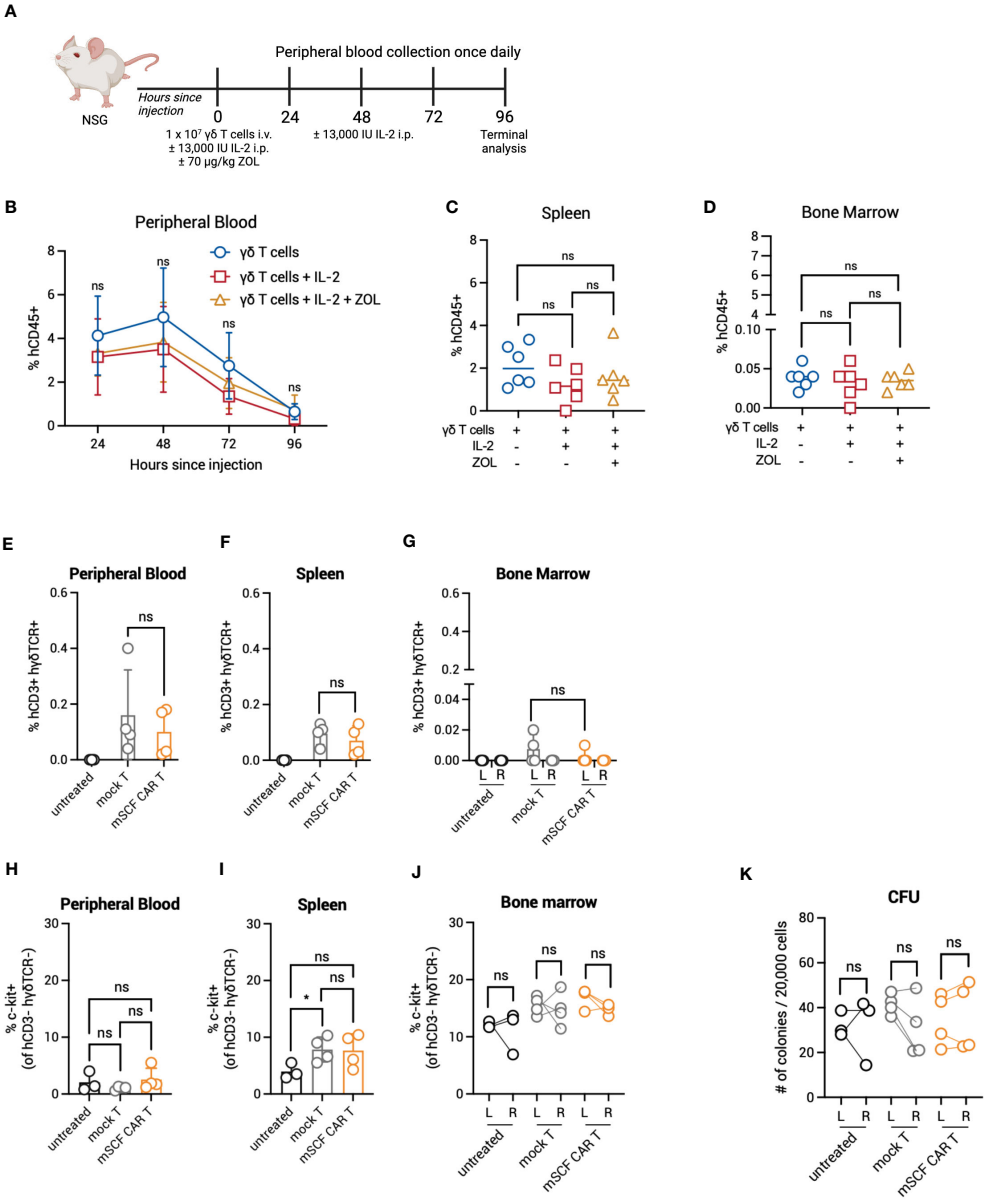
Persistence and toxicity of mSCF CAR γδ T cells in immunocompromised mice. **(A)** Schematic. NSG mice were injected with 1 × 10^7^ γδ T cells IV alone on day 0 (n = 6), 1 × 10^7^ γδ T cells IV on day 0 and two doses of 13,000 IU IL-2 IP on day 0 and day 2 (n = 6), or 1 × 10^7^ γδ T cells IV on day 0, two doses of 13,000 IU IL-2 IP on day 0 and day 2, and one dose of 70 μg/mg zoledronic acid SC on day 0 (n = 6). Peripheral blood leukocytes were collected daily beginning 24-hours after the start of treatment and assessed for the presence of human γδ T cells. Four days later, mice were sacrificed to assess for human γδ T cells within the spleen and bone marrow. **(B)** %hCD45+ (gated on live cells) within the peripheral blood at each timepoint. Error bars represent SD. Statistical analysis represents Student’s t test (ns, p > 0.05). **(C–D)** %hCD45+ (gated on live cells) within the spleen **(C)** and bone marrow **(D)** at end point. Statistical analysis represents Student’s t test (ns, p > 0.05). **(E–G)** %hCD3+ hγδTCR+ (gated on live cells) within the peripheral blood **(E)**, spleen **(F)**, and the left and right femurs **(G)**. Error bars represent SD. Statistical analysis represents Student’s t test (ns, p > 0.05). **(H–J)** %c-kit+ (gated on hCD3- hγδTCR- live cells) within the peripheral blood **(H)**, spleen **(I)**, and left and right femurs **(J)**. Error bars represent SD. Statistical analysis represents unpaired **(H, I)** or paired **(J)** Student’s t test (*p < 0.05; ns, p > 0.05). **(K)** Number of colonies counted from a CFU assay on the left and right femurs. Statistical analysis represents paired Student’s t test (ns, p > 0.05). Data point graphed is averaged from n = 3 technical replicates per mouse.

Given enhanced toxicity of mSCF CAR γδ T cells *ex vivo*, and the *in vivo* expansion of αβ T cells in murine bone marrow, we next determined γδ T-cell kinetics *in vivo* to evaluate possible bone marrow toxicity. To this end, NSG mice were injected with 10^7^ γδ T cells by intravenous, retro-orbital injection and peripheral blood leukocytes were collected once daily for 4 days (**Figure 7A**). To determine if IL-2 or zoledronic acid enhanced *in vivo* persistence, a treatment regimen of two doses of 13,000 IU recombinant human IL-2 by intraperitoneal injection and one dose of 70 μg/kg zoledronic acid by subcutaneous injection or two doses of IL-2 alone were given. Percent γδ T cells were highest in circulation 48 hours after injection, with a steady decline in persistence up to 4 days after injection (**Figure 7B**). γδ T cells were found in the spleen (**Figure 7C**) and bone marrow 4 days after injection, though very low levels were found in the bone marrow (**Figure 7D**). Neither treatment with IL-2 or combination IL-2 and zoledronic acid treatment enhanced γδ T-cell persistence.

The lack of γδ T cells infiltrating the extravascular bone marrow space of NSG mice may prove advantageous to control on-target off-tumor toxicity in the bone marrow compartment. To investigate this further, we directly injected mSCF CAR γδ T cells into the left femur of NSG mice, with mock γδ T cells as a control, and assessed for bone marrow clearance two days later, when γδ T cells have previously been shown to be at their highest in circulation. Indeed, human CD3^+^ human γδTCR^+^ γδ T cells were found in the peripheral blood and spleen two days after injection (**Figures 7E–G**). However, only a small number γδ T cells remained in the injected left femur (**Figure 7G**). Additionally, changes in the c-kit^+^ compartment of the peripheral blood, spleen, and bone marrow of the left and right femur were not observed between groups (**Figures 7H–J**). A CFU assay confirmed no changes in progenitor cell populations between the left (injected) and right (control) femur (**Figure 7K**).

Together, these data show γδ T cells do not persist beyond four days in NSG mice and do not efficiently infiltrate the extravascular bone marrow compartment. This, in addition to transient transgene expression, can serve as a safety mechanism to limit toxicity.

### 3.7 hSCF sBite-modified γδ T cells moderately improve survival *in vivo*

**Figure 8:**
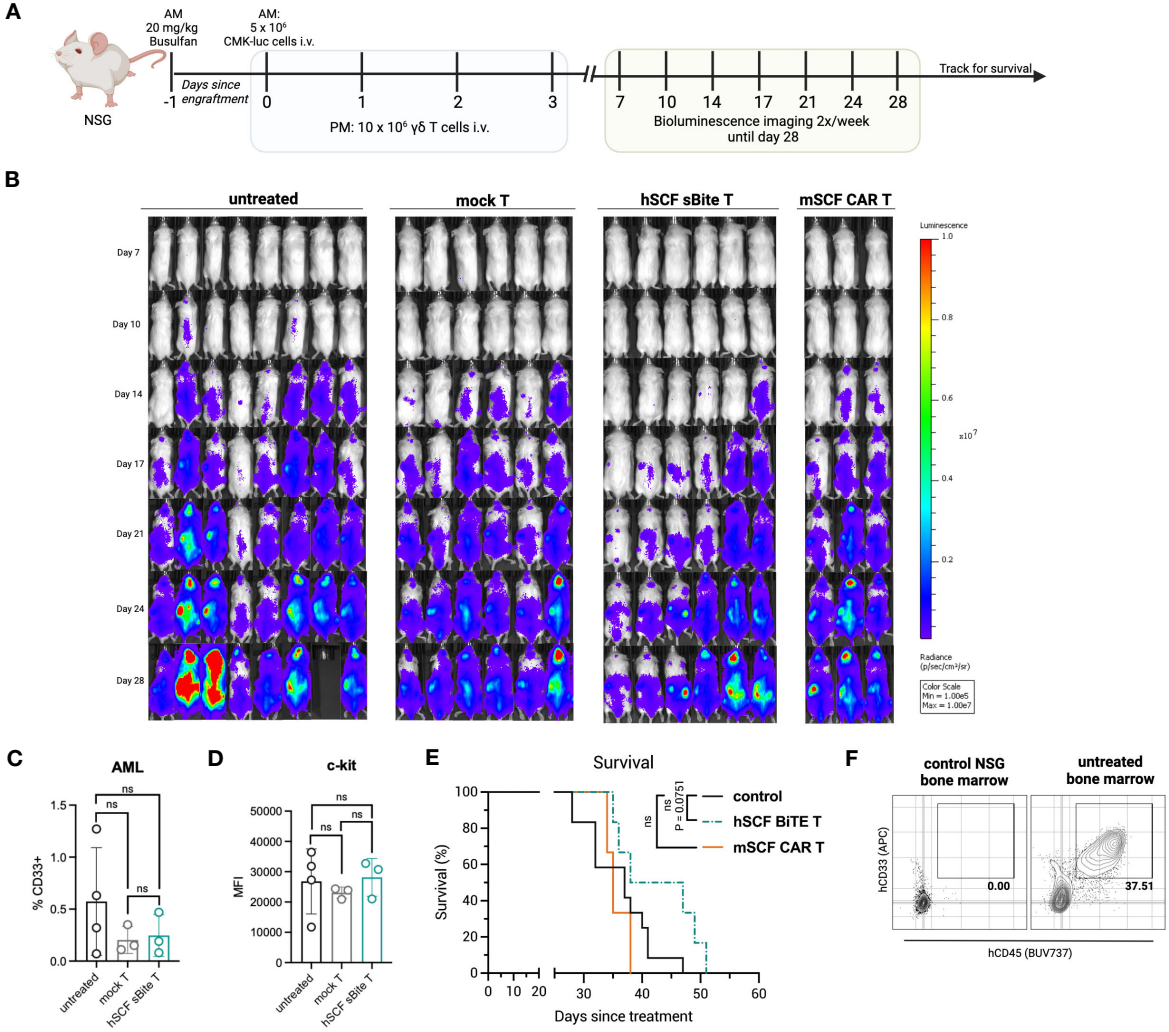
Treatment of hSCF sBite-modified γδ T cells only moderately prolongs survival *in vivo*, despite aggressive treatment regimen. **(A)** Experimental design. Briefly, NSG mice were pre-conditioned with 20 mg/kg busulfan IP on day -1, then injected with 5 × 10^6^ CMK cells via tail-vein injection in the morning on day 0. Beginning in the afternoon on day 0, and then once daily for the next 3 days for a total of 4 doses, 1 × 10^7^ γδ T cells were injected via tail-vein injection. Mice were subjected to bioluminescence imaging for the following 3 weeks, then followed for survival until they met endpoint. n = 8 untreated, n = 6 mock T treated, n = 6 hSCF sBite treated, n = 3 mSCF CAR treated. **(B)** Bioluminescence images. **(C)** Peripheral blood leukocytes were collected 3 weeks after the start of treatment and assessed for presence of CD33^+^ CMK cells. Error bars represent SD. Statistical analysis represents Student’s t test (ns, p > 0.05). **(D)** MFI of c-kit on CMK cells within the periphery. Statistical analysis represents Student’s t test (ns, p > 0.05). **(E)** Kaplan-Meier survival analysis. Untreated and mock γδ T treated groups were combined as a control group. Statistical analysis represents log rank (Mantel-Cox) test (ns > 0.05). P-value is shown. **(F)** Representative flow plots of hCD33^+^ hCD45^+^ CMK cells in the bone marrow of an untreated mouse sacrificed near end-point.

To assess efficacy against AML *in vivo*, NSG mice were first pre-conditioned with busulfan, then 5 × 10^6^ luciferase-expressing CMK cells were established by intravenous injection the following morning. Beginning that afternoon, and for the next 3 days for a total of 4 doses, 1 × 10^7^ total mSCF CAR γδ T cells or hSCF sBite γδ T cells were injected intravenously, with mock γδ T cells as a control (**Figure 8A**). Bioluminescence was assessed regularly throughout the study (**Figure 8B**).

There was a clear delay in cancer development in hSCF sBite γδ T cell-treated mice that was not observed in mSCF CAR γδ T cell-treated mice (**Figure 8B**). We postulate this suppression of growth despite similar *in vitro* results can be attributed to the secretion of the hSCF sBite and its ability to engage all γδ T cells, whereas the mSCF CAR can only function through transfected cells as it is a surface-bound protein.

Three weeks after the establishment of AML, peripheral blood leukocytes were collected and subject to flow cytometry analysis for the presence of CD33^+^ CMK cells. At this time, there were few CMK cells in the peripheral blood, especially in mSCF CAR and hSCF sBite treated mice (**Figure 8C**). The mean percentage of CMK cells was lower in CAR and sBite treated mice compared to untreated mice (0.3% vs 0.6%), however the differences did not approach significance (**Figure 8C**). The surface expression of c-kit, as measured by MFI, on circulating CMK cells did not differ among groups, suggesting antigen escape is not a mechanism of immune evasion (**Figure 8D**). Complete blood counts (CBCs) did not show significant differences among groups, though all mice experienced thrombocytopenia, which was likely due to cancer progression affecting hematopoiesis (**Figures S4A–H**).

Treatment with mSCF CAR did not result in a significant survival benefit under the conditions tested, whereas hSCF sBite γδ T cells showed a trend towards survival enhancement (**Figure 8E**, *p* = 0.075). One possibility as a mechanistic explanation for the discrepancy between the efficient *in vitro* cytotoxicity and *in vivo* tumor clearance is the observation that surviving CMK cells were primarily observed in the extravascular bone marrow compartment (**Figure 8F**). We have previously shown that γδ T cells do not readily migrate to murine bone marrow (39), possibly due to minimal CXCR4 expression (**Figure S5**), an important chemokine receptor that helps leukocytes migrate from the periphery to the bone marrow (51). Taken together, these data suggest CMK cell growth is inhibited at early time points when injected into peripheral circulation up until they leave circulation and populate the bone marrow, where even high doses of γδ T cells cannot control disease burden in the extravascular bone marrow compartment.

## 4 Discussion

AML proves more difficult to manage with targeted therapeutics, such as CAR T therapy, than B-cell malignancies due to a scarcity of cancer-specific target receptors. Despite this, preclinical efforts to develop CAR T therapies for AML are ongoing and include targets such as CD33 (14), CD123 (52, 53), and CD70 (54). Most notably, therapeutics targeting CD33 (NCT03971799) and CD123 (NCT04678336) have advanced to clinical trials and show some clinical promise. However, critical measures are needed to mitigate off-target toxicities of these therapies within the bone marrow niche, as HSCs express both CD33 and CD123. As HSCT is curative for patients with AML and toxicities associated with pre-transplantation conditioning are severe (8), it has been observed that these toxicities may be advantageous and can even replace pre-transplantation conditioning.

C-kit, or CD117, has also been explored as a potential target for the development of CAR T therapy in the context of AML (22, 55). As c-kit is expressed on HSCs, current therapeutics targeting c-kit have been designed as a bridge-to-transplant by eradicating HSCs and leukemia simultaneously. Indeed, preclinical studies targeting c-kit as a means of pre-transplantation conditioning alone using antibody-immunotoxin conjugates by our lab (17) and others (18) and using CAR T cells (21) are ongoing. Clinically, briquilimab has shown promise in a Phase 1/2 dose-escalation trial of patients undergoing HSCT for severe combined immunodeficiency (SCID; NCT02963064) and a Phase 1 trial of patients undergoing HSCT for MDS/AML (NCT04429191).

Herein, we designed a ligand-based CAR and sBite to target c-kit using its cognate ligand SCF for the treatment of AML. The reasons for choosing a ligand-based design over a traditional scFv are many. Primarily, the elimination of stabilizing interactions that exist in the original antibody structure and simultaneous introduction of a non-native linker sequence between the variable heavy and light domains can result in thermal instability, partial scFv unfolding, domain swapping, and uncontrolled heterodimerization between two scFv molecules (27, 56). Therefore, to ensure stability of the antigen-binding domain and prevent scFv aggregation leading to tonic signaling of the CAR or inefficiency of the sBite, we tested a ligand-based design. It is true that some ligands, such as SCF, have dimerization potential; however, this does not interfere with receptor binding (and, in turn, therapeutic efficacy), as ligand dimers may still bind their receptors, though it may lead to enhanced tonic signaling. In fact, this design may prove advantageous over a traditional scFv-based design due to differences in affinity of the binding domain to the receptor. While the binding affinity for human SCF to human c-kit is strong and falls within the nanomolar range (57), it is still weaker than that of an antibody-antigen interaction, which can fall as low as picomolar range (58). Recent studies suggest that low-affinity CAR T cells have a lower risk of antigen escape through decreased trogocytosis (59), increased tumor selectivity (60), reduced exhaustion (61), and a reduced risk of cytokine release syndrome (CRS) due to decreased cytokine secretion (61). Together, these benefits may promote an enhanced safety profile of the therapeutic and allow for the potential combination with other therapeutics. That said, ligand-based therapeutics have been tested preclinically (62), with some advancing to clinical testing. For example, ligand-based IL-13Rα2-targeting CAR T cells whose binding domain utilizes a modified IL-13 ligand have shown promise for the treatment of childhood glioblastoma multiforme (GBM), resulting in tumor regression with enhanced IFN-γ signaling to activate the host immune system (63, 64).

The strategy of expressing CAR or sBite transgenes transiently in γδ T cells (rather than traditional αβ T cells) can serve as a safety modification and as a potential off-the-shelf therapeutic. γδ T cells do not cause graft-versus-host-disease (GvHD) reactions when transplanted across HLA barriers, as γδ T cells recognize antigen independent of HLA, while also contributing to graft-versus-leukemia (GvL) effect partially through the recognition of stress antigens (29–31). Importantly, pediatric patients with higher numbers of γδ T cells after HSCT have significantly higher event-free survival, a lower probability of infection, and a lower risk of relapse, with the survival advantage lasting as long as 7 years after HSCT (65, 66). Additionally, γδ T-cell infiltration into tumors has been identified as the most favorable prognostic factor in a pan-cancer analysis (67), further solidifying their clinical benefit.

We have been able to expand γδ T cells *ex vivo* from healthy donors using a serum-free Good Manufacturing Practice (GMP) compliant expansion protocol (33, 35) and have shown preclinical efficacy in B-cell leukemia (39) and neuroblastoma models (32, 38). In fact, this manufacturing strategy is under clinical investigation in a Phase 1 trial in the context of childhood neuroblastoma (NCT05400603). With respect to AML, it is known that difficulties in manufacturing multiple doses and a lack of persistence of mRNA-based αβ CAR T cells led to limited outcomes in clinical trials for anti-CD123 mRNA αβ CAR T cells in patients with r/r AML (NCT02623582) (68). Concerns about manufacturing can be reduced by instead employing γδ T cells as opposed to αβ T cells, which can be manufactured from healthy donors and serve as an off-the-shelf therapeutic. Furthermore, transient expression of a c-kit-directed therapeutic may limit off-target toxicity in the bone marrow niche, and un-modified γδ T cells can provide additional cancer surveillance through interaction with stress antigens, which we have shown are up regulated on AML cells.

In this study, c-kit-directed, ligand-based, mSCF CAR and hSCF sBite γδ T cells were generated by mRNA electroporation, achieving >60% CAR modification on average. *In vitro*, we show mSCF CAR- and hSCF sBite-modified γδ T cells are cytotoxic against c-kit^+^ AML cell lines, ablating >90% of AML cell lines during a short-term cytotoxicity assay at a low effector-to-target ratio. Furthermore, a significant decrease in the LSK compartment of murine bone marrow during a 24-hour *ex vivo* co-culture was shown with mSCF CAR-modified γδ T cells, confirming the possibility of toxicity within the bone marrow compartment. Interestingly, this decrease in the LSK compartment was observed in both the absence and presence of supraphysiological levels of murine SCF and was confirmed by CFU assay, suggesting mSCF CAR γδ T cells affected LSK pluripotency. One possible explanation for this surprising result is that while the addition of SCF to the co-culture may have initially protected LSK cells from γδ T-cell induced cell death by c-kit receptor internalization, subsequent re-expression of c-kit on the cell surface 12-14 hours later (69) may render them susceptible again, though more experiments are needed to test this hypothesis.

However, despite this toxicity *ex vivo*, it was not observed *in vivo*, likely due to limited trafficking of γδ T cells to the bone marrow niche. As such, treatment of AML-bearing mice with hSCF sBite-modified γδ T cells only slightly prolonged survival. This, in combination with previously published (38, 39, 70) and ongoing studies within our group, now defines an important limitation of studying human IL-2/zoledronic acid-expanded γδ T cells in the murine setting, as these cells do not migrate to the bone marrow, which is the site of later stage CMK cell growth in this AML mouse model. Alternatively, it is also possible that the inefficient migration of human γδ T cells into the murine extravascular bone marrow compartment may simply be due to specific-specific chemokine-receptor incompatibility. Despite this limitation, our data suggest that the hSCF sBite provides better control of tumor growth than the mSCF CAR, which can be attributed to the activation of both modified and non-modified cells, as the sBite is secreted and can interact with all CD3^+^ cells.

As many myeloid malignancies include bone marrow involvement, a lack of trafficking of γδ T cells to the bone marrow niche proves challenging for clinical translation. Indeed, others have shown difficulty in trafficking of leukocytes to the bone marrow and have proposed the overexpression of CXCR4 by retroviral transduction as a means of enhancing trafficking (21). Another strategy may instead aim to enhance CXCR4 expression through modification of our expansion protocol, as it has been shown that the addition of TGF-β to expansion media upregulates CXCR4 on γδ T cells (70, 71). However, to date, we have not been successful in repeating this finding, possibly due to our unique serum-free method of expansion. Further studies aim to enhance γδ T-cell persistence in the bone marrow by similar modifications, and are currently underway. Despite these limitations, these data demonstrate ligand-based γδ T-cell therapies targeting c-kit for the treatment of AML is fundamentally possible. Future investigation will focus on efforts to enhance γδ T cell migration and infiltration into the bone marrow, and specifically address toxicities associated with hSCF sBite expression within the bone marrow compartment.

## Data availability statement

The raw data supporting the conclusions of this article will be made available by the authors, without undue reservation.

## Ethics statement

The studies involving humans were approved by the Institutional Review Board (IRB), Emory University, Atlanta, GA. The studies were conducted in accordance with the local legislation and institutional requirements. The participants provided their written informed consent to participate in this study. The animal study was approved by the Institutional Animal Care and Use Committee (IACUC), Emory University, Atlanta, GA. The study was conducted in accordance with the local legislation and institutional requirements.

## Author contributions

GB: Conceptualization, Data curation, Formal Analysis, Funding acquisition, Investigation, Methodology, Visualization, Writing – original draft, Writing – review & editing. JL: Investigation, Writing – review & editing. JO: Investigation, Writing – review & editing. KP: Investigation, Writing – review & editing. JA: Investigation, Writing – review & editing. RA: Investigation, Writing – review & editing. AF: Resources, Writing – review & editing. BY: Resources, Writing – review & editing. DM: Resources, Writing – review & editing. HB: Resources, Writing – review & editing. SC: Conceptualization, Methodology, Writing – review & editing. BP: Conceptualization, Methodology, Writing – review & editing. CD: Conceptualization, Data curation, Formal Analysis, Funding acquisition, Methodology, Supervision, Writing – review & editing. HS: Conceptualization, Data curation, Formal Analysis, Funding acquisition, Methodology, Supervision, Writing – review & editing.
