# Supplementary material for: Ligand-based targeting of c-kit using engineered γδ T cells as a strategy for treating acute myeloid leukemia
Source: Front Immunol. 2023 Nov 13;14:1294555. doi: 10.3389/fimmu.2023.1294555 (PMC10679681; doi:10.3389/fimmu.2023.1294555)
Supplement: Supplementary file 1 [file DataSheet_1.pdf]

Supplementary Material

Ligand-based targeting of c-kit using engineered γδ T cells as a strategy for treating acute myeloid leukemia

Gianna M. Branella[1,2,3], Jasmine Y. Lee[1,2,3], Jennifer Okalova[1,2,4], Kiran K. Parwani[1,2,3], Jordan S. Alexander[1,3], Raquel F. Arthuzo[1,2,3], Andrew Fedanov[1,3], Bing Yu[5], David McCarty[5], Harrison Brown[5], Shanmuganathan Chandrakasan[1,3], Brian G. Petrich[5], Christopher B. Doering[1,3,4], H. Trent Spencer[1,3,4*]

[1]Department of Pediatrics, Emory University School of Medicine, Atlanta, GA, United States

[2]Cancer Biology Program, Graduate Division of Biological and Biomedical Sciences, Laney Graduate School, Emory University, Atlanta, GA, United States

[3]Aflac Cancer and Blood Disorders Center, Children's Healthcare of Atlanta, Atlanta, GA, United States

[4]Molecular Systems Pharmacology Program, Graduate Division of Biological and Biomedical Sciences, Laney Graduate School, Emory University, Atlanta, GA, United States

[5]Expression Therapeutics, Inc., Tucker, GA, United States

*** Correspondence:**
H. Trent Spencer
hspence@emory.edu

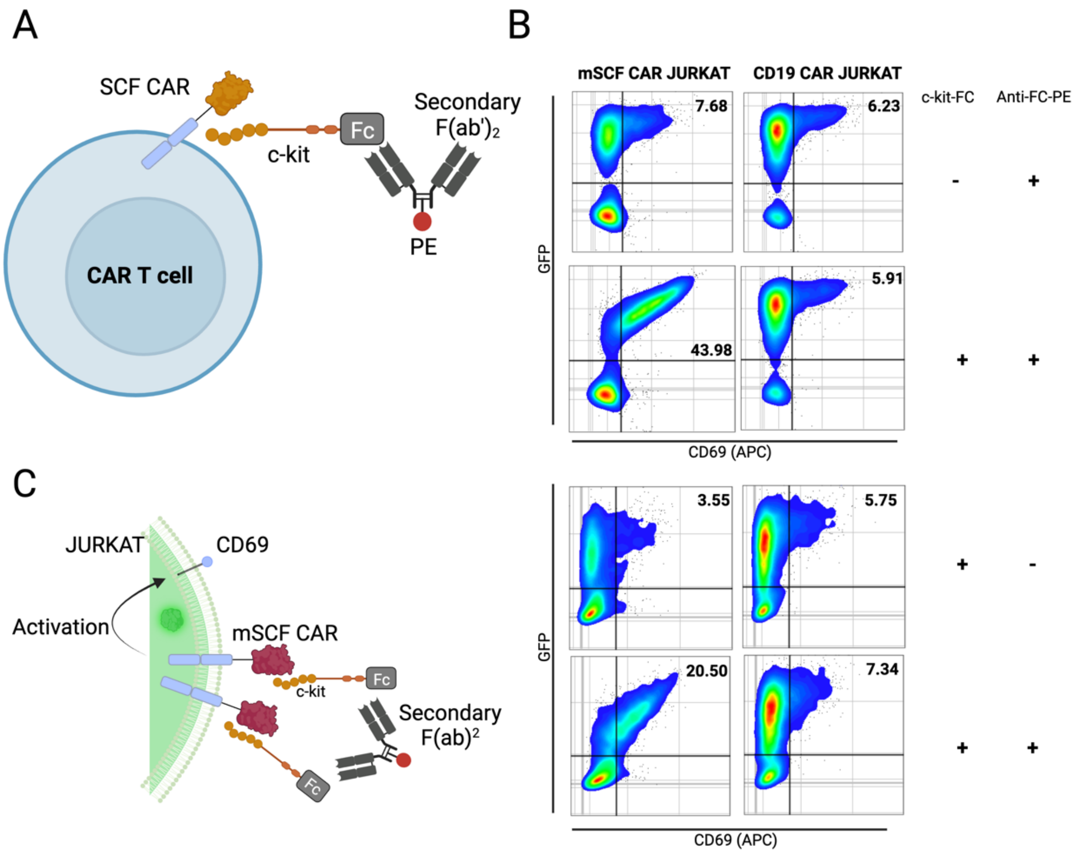

Supplementary Figure 1. CAR detection protocol activates Jurkat T cells. (A) Schematic of standard CAR staining protocol. (B) Representative flow cytometry plots depict CD69+ activation of CAR+ cells when both the c-kit-Fc chimera and secondary detection F(ab')$_2$ are incubated with CAR+ cells. (C) Possible explanation for CAR+ activation in (B).

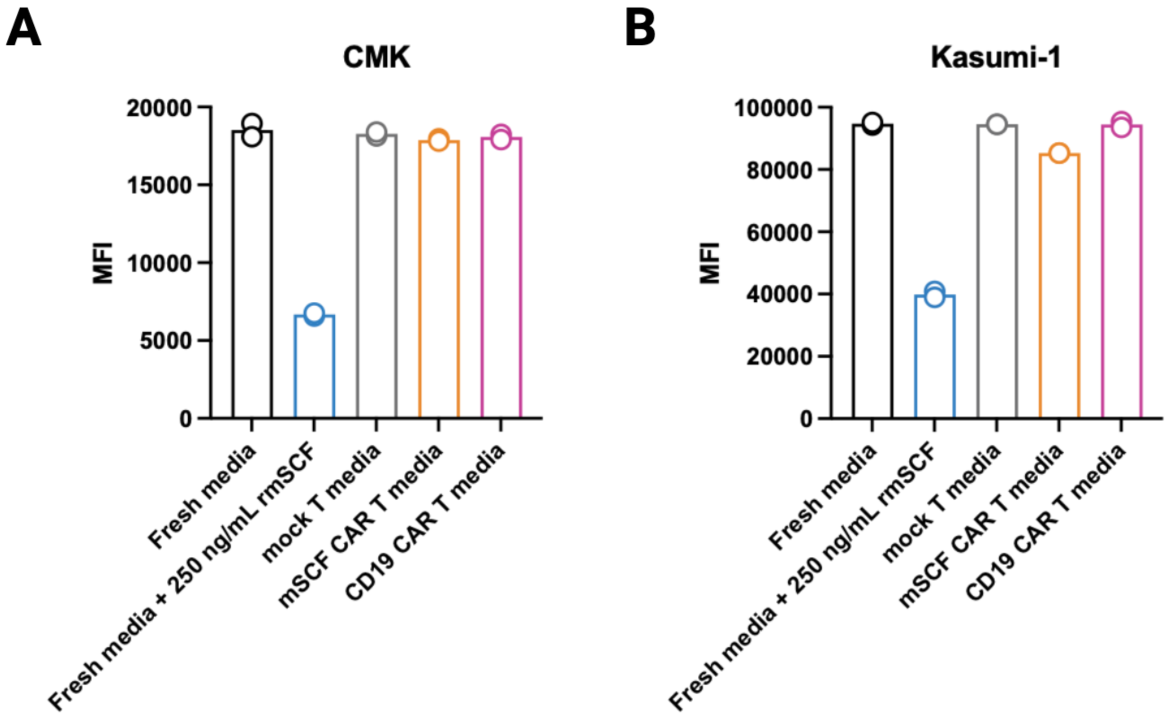

Supplementary Figure 2. mSCF CAR is not shed from the surface of modified αβ T cells. Briefly, c-kit+ AML cell lines CMK (A) or Kasumi-1 (B) were cultured with fresh T cell media or media that has been conditioned with mock T cells, mSCF CAR T cells, or CD19 CAR T cells. Fresh media with 250 ng/mL recombinant murine SCF was spiked in as a control. c-kit MFI is graphed. n = 2 experimental replicates.

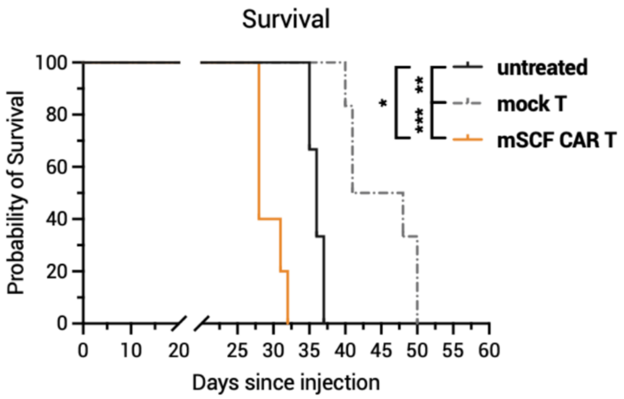

Supplementary Figure 3. Treatment with mSCF CAR αβ T cells adds significant toxicity. Kaplan-Meier survival analysis. Statistical analysis represents log rank (Mantel-Cox) test (***$p < 0.001$; **$p < 0.01$; *$p < 0.05$).

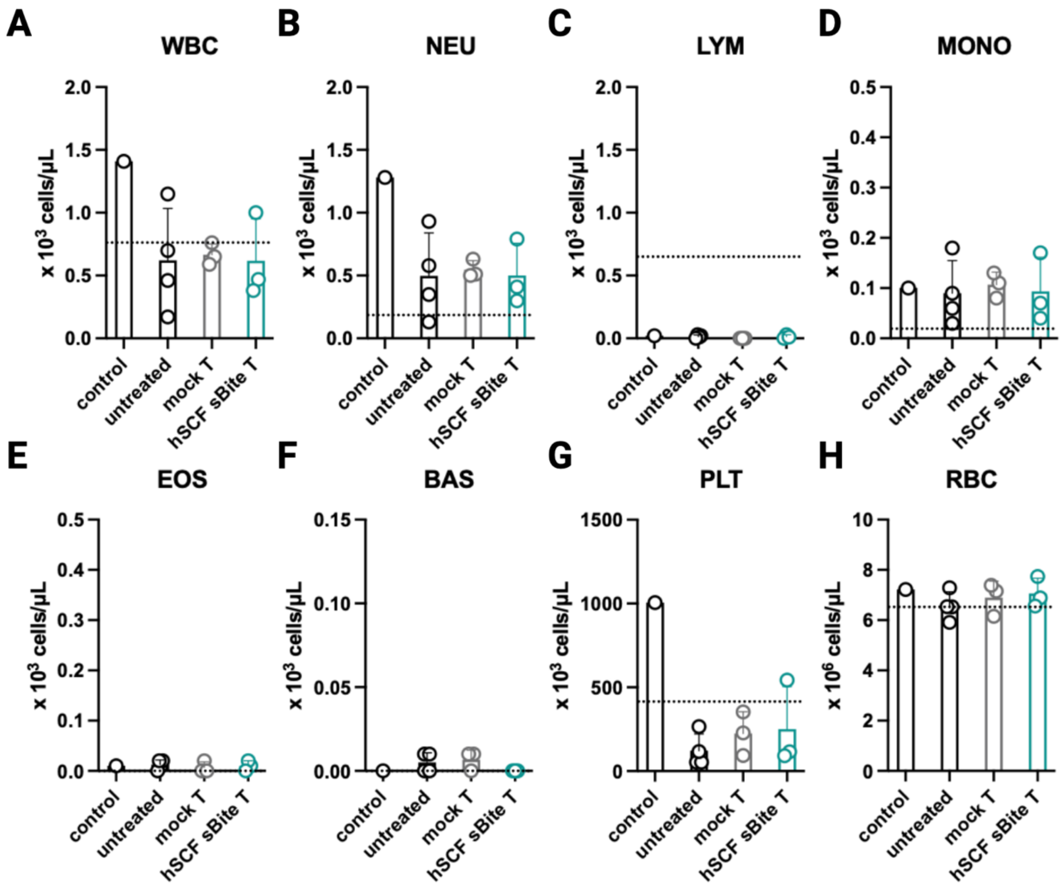

Supplementary Figure 4. Complete blood counts do not change with mock γδ T or hSCF sBite γδ T treatment. White blood cell (A), neutrophil (B), lymphocyte (C), monocyte (D), eosinophil (E), basophil (F), platelet (G), and red blood cell (H) counts for control NSG (n = 1), untreated (n = 4), mock γδ T treated (n = 3), and hSCF sBite γδ T treated (n = 3) mice from Figure 8.

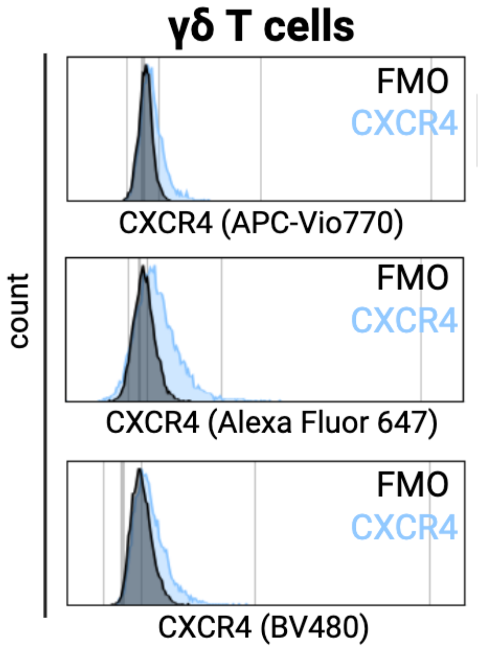

Supplementary Figure 5. γδ T cells do not express CXCR4. Histograms of CXCR4 expression on cultured γδ T cells with 3 different CXCR4 antibodies (top: clone REA649, middle: clone 44716, bottom: clone 12G5).
